# Firework aversion in cats and dogs as reported by Dutch animal owners

**DOI:** 10.1016/j.vas.2024.100402

**Published:** 2024-10-16

**Authors:** Ineke R. van Herwijnen, Claudia M. Vinke, Saskia S. Arndt, Pascalle E.M. Roulaux

**Affiliations:** Division of Animals in Science and Society, Faculty of Veterinary Medicine, Department of Population Health Sciences, Utrecht University, Utrecht, The Netherlands

**Keywords:** Dogs, Cats, Firework aversion, Firework fear, Firework phobia, Noise sensitivity

## Abstract

Firework (noise) aversion is seen in cats and dogs relatively often and comes with a risk of animal welfare impairment. Affected animals may show behaviours like freezing, hiding, fleeing, but also inappropriate elimination, loss of appetite and (social) withdrawal. In The Netherlands, firework regulation has been a much-debated topic and we aimed to provide insight into how owners in this country report on their cat's or dog's firework aversion. We gathered reports on 3,009 dogs’ and 622 cats’ firework-related behaviours, exposure, animal (early life) characteristics and owner (dis)agreement with statements relating to firework aversion and animal guidance. We hypothesized that 1) suboptimal early life factors come with higher prevalence of firework aversion and 2) owners that report less firework aversion for their animal, agree more to the statement that owner behaviour affects an animal's future reactions to firework. Our findings confirm the first hypothesis for dogs and the second hypothesis for both species. Firework affects Dutch cats, dogs and their owners not just on New Year's Eve, but for multiple months a year: 64 % of the owners heard firework noise from September onwards. Suboptimal early life factors, of for instance not being noise habituated as a young animal came with higher prevalence of firework fear for dogs, but not cats and habituating particularly kittens seemed uncommon (4 % of kittens). Advice seeking was more common for dogs (54 %) than cats (23 %) with firework aversion. For both species reporting of lasting effects of therapeutic interventions was uncommon. <30 % of the most often applied interventions were reported to have a lasting mitigating effect on firework aversion. This may indicate a high importance of hereditary and early life preventive measures, but our respondents were not in high agreement with statements reflecting such importance. Although our study presents associative evidence only and was based on owner report, not on direct animal observations, we present attention points for future studies. One such attention point is a seemingly low awareness of the lifelong influence of an animal's early life factors. Studying how such awareness affects people that opt for a puppy or kitten as to facilitate acquisition decisions that may or may not come with firework resilient animals, may ultimately benefit animals that were in this study seen to experience firework aversion for multiple months a year, with limited long-term effectiveness of therapeutic interventions.

## Highlights

• Firework affects Dutch cats, dogs and their owners for multiple months a year.
• Noise habituation of a puppy related to less reporting of adult dog firework fear.
• Owner reporting on animal guidance related to animal reactions to firework.

## 1 Introduction

Noise aversion is common in cats and dogs (Blackwell et al., 2013; Ballantyne, 2019; Salonen et al., 2020) and defined as an anxious, fearful or phobic response to noise (Ballantyne, 2019). Noise sensitivity is an alternative terminology used for noise aversion (Storengen & Lingaas, 2015; Tiira et al., 2016). The severity of such an aversion varies between individual animals. The more severe cases of noise aversion lead to an animal suffering from stress, anxiety and/ or fear (Blackwell et al., 2013). Anxiety and fear are regarded as adaptive behavioural, emotional and physiological responses (Levine, 2008; Blackwell et al., 2013). Anxiety reflects a negative emotional state, brought about by the environment or certain elements within it, that to the animal predict a possible threat (Levine, 2008; Ohl et al., 2008; Blackwell et al., 2013; Tiira et al., 2016; Ballantyne, 2019). Fear reflects a protective response to an actual or perceived threat (Levine, 2008; Ohl et al., 2008; Blackwell et al., 2013; Tiira et al., 2016; Ballantyne, 2019). As a contrast to anxiety and fear, phobia is regarded as a non-adaptive, extreme, long lasting, or overly intense reaction to a (perceived) threat (Levine, 2008; Ohl et al., 2008; Blackwell et al., 2013). Thus, noise aversion can range from functional fear responses in animals to more severe signs of suffering from noise anxiety or even phobia. Owners may report an array of behaviours when indicating their cat's or dog's noise aversion, which are not necessarily unique to *noise* aversion. The behaviours may be seen in other aversive experiences than of noise aversion (Gähwiler et al., 2020). Examples of reported noise aversion behaviours are meowing or barking, clinging to a familiar human, crouching, destruction, escaping confinement, freezing, hiding, inappropriate elimination, loss of appetite, pacing, panic (fleeing without considering environmental barriers, such as doors or windows), panting, restlessness, salivating, shaking, social withdrawal, trembling (Dale et al., 2010; Blackwell et al., 2013; Tiira et al., 2013; Ballantyne, 2019; Gates et al., 2019; Gähwiler et al., 2020).

Prevalence of noise aversion is reported at varying levels in animals (Fatjo et al., 2006; Blackwell et al., 2013; Storengen & Lingaas, 2015; Tiira et al., 2016; Gates et al., 2019; Riemer, 2019; Salonen et al., 2020). In a representative sample of Danish dog owners, noise was the most common source of a fear problem (Meyer et al., 2023). Dale and colleagues (2010) reported 46 % of 3527 cats and dogs to exhibit signs of fear in response to firework (*N* = 1635), with the fearful dogs (*N* = 684) described as most often exhibiting shivering/ trembling (74 % of fearful dogs) hiding (71 %), and cowering (47 %). The fearful cats (*N* = 951) were reported to most often hide (85 % of fearful cats), escape/ run away (46 %), and shiver/ tremble (40 %) or cower (39 %; Dale et al., 2010). The duration of fear responses was not seen to differ between cats and dogs (Dale et al., 2010). Yet, a difference between these two species was seen particularly for vocalisations. Dogs vocalised more than cats, with an estimated occurrence of the behaviour in dogs relative to cats of 2.3 (CI: 1.9–2.9, *P* = 0.0001; Dale et al., 2010). Variation in the prevalence of noise aversion may be based in part on the composition of the study population. However, which type of noise is surveyed, may also matter. Noise aversion regarding specifically firework, seems to be reported on relatively often (Blackwell et al., 2013; Storengen & Lingaas, 2015). This may indicate strong aversiveness of this type of loud noise. Alternatively, the high report levels for firework aversion may (also) be due to firework encompassing more than noise only. This as firework can come with olfactory and visual stimuli.

Firework aversion coincided with aversion to other loud noise sources, such as of guns and thunder (Dale et al., 2010; Blackwell et al., 2013; Storengen & Lingaas, 2015; Tiira et al., 2016; Gates et al., 2019). Whereas general noise aversion, including for instance traffic noise, was seen to coincide with other anxieties or fears in dogs (Blackwell et al., 2013). Hereditary factors and early life experiences may create vulnerability in animals to noise aversion. Noise sensitivity is in part hereditary and a lack of habituation to noise during socialisation phases will affect the chances of an animal developing noise aversion (Casey & Bradshaw, 2008; Storengen & Lingaas, 2015; Ballantyne, 2019; Salonen et al., 2019, 2020). Particularly loud noise, such as firework noise, through its loudness, suddenness and with the animal experiencing no control or predictability over its occurrence, can create mental trauma in dogs and cats, with consequences for both animal welfare and companion animal ownership satisfaction (Storengen & Lingaas, 2015; Ballantyne, 2019; Salonen et al., 2020). Consequences for the animal may regard mental and physical health decline, with some (dog) owners reporting recovery from firework to take up to a week or even longer (Riemer, 2019). An example of a direct physical health consequence of noise aversion, was in reported appetite loss in dogs confronted with acoustic stress due to football matches (Carrierri-Rocha et al., 2020). Furthermore, although noise aversion may be stimulus specific, and for instance at first be triggered by firework only, through the process of generalisation, the anxiety, fear or phobia may be experienced by an animal in an increasingly broad set of contexts and triggered by an increasingly broad set of stimuli (Ballantyne, 2019).

As mentioned before, especially firework may cause strong noise aversion in cats and dogs (Blackwell et al., 2013), which may be explained by the particular loudness of the involved noise (Kukulski et al., 2018), that may be paired with additional (olfactory and visual) sensory input. Also, firework is likely experienced as an unpredictable and uncontrollable element of an animal's environment, which comes with animal welfare risks (Ohl & Van der Staay, 2012). How often firework and thus these animal welfare risk increasing elements are in the environment, may differ between countries. Policies and customs on firework vary between countries, likely leading to differences in exposure of animals to firework as a potential source of noise aversion. In The Netherlands firework regulation has for many years been a source of debate and owners may vary in their stance on firework regulation and their opinion on how to guide animals with noise aversion. This indicates how firework affects humans as well as animals. Yet, for cats and dogs in The Netherlands, no scientific information is presently available to provide insight on how these animals’ owners report on their animals being affected by firework, if the aforementioned animal early life factors come with higher firework aversion prevalence and if the aforementioned varying owner opinions on how to guide animals with noise aversion are related to reported severeness of animal firework aversion.

Hence, we aimed to study how Dutch cats, dogs and their owners are affected by firework noise and how owners sought and experienced possible solutions for their animal's firework (noise) aversion. In addition to these descriptives, we tested two hypotheses. Firstly we hypothesized that suboptimal early life factors come with higher firework aversion prevalence. Secondly, we predicted that owners of less firework aversive animals, opinion more strongly that owner reactions determine future animal firework reactions. If both hypotheses are confirmed, this study may lay a basis for future studying of how owner opinions and choices may factor into helping animals become resilient to development of firework aversion, such as through early life factor optimalisation.

## 2 Methods

### 2.1 Ethical considerations

The online questionnaire introduction explained the purpose of the research and the study did not involve treatments or interventions in the life of respondents or their dogs. The questionnaire was not repeated, meaning it did not interfere significantly with normal daily life and did not include questions that were psychologically stressful. The study was approved by the Ethics Committee of the Faculty of Social and Behavioural Sciences of Utrecht University on September 1st, 2023.

Informed consent was not obtained as respondents chose to participate freely via internet and the purpose of the research was stated at the start of the online questionnaire and after this, respondents needed to actively state their wish to participate or decline from participating. Data was gathered anonymously and did not include any privacy sensitive information, such as income or educational background of the human respondents.

### 2.2 Questionnaire

Reports by cat and dog owners were used to evaluate how Dutch cats, dogs and their owners are affected by firework noise and how owners sought and experienced possible solutions for their animals’ firework aversion. We collected data via an online questionnaire in Qualtrics®. A convenience sample of respondents was recruited via the internet, including websites frequently visited by cat and dog owners, internet fora on cat and dog topics, social media channels from Utrecht University and other organisations, such as animal protection organisations, newspaper, and television interviews. The questionnaire data was gathered from the 12th of October 2023 until the 26th of November 2023. Anyone aged 18 years and over, responsible for at least 50 % of the care for a cat or dog was eligible for the research and we did not practice (other) criteria for inclusion or exclusion.

Details on the participating cat owners (*N* = 622) and dog owners (*N* = 3009) and their animals are presented in the results section. The questionnaire was in Dutch, but see Appendix A for the English translation, and consisted of four sections of owner opinions on animal interactions, the animal, its living environment, its early life experiences, firework exposure and firework reactions respectively. Questions on for instance the animal's firework exposure were mandatory. However, as to engage a high number of respondents, we made questions facultative on for instance a possible treatment of firework (noise) aversion.

We found no validated questionnaire in scientific literature that could serve as a basis for our explorative study. Where possible we based our questionnaire on previous literature, for example to establish the list of fear and stress signs and possible interventions on the animal's noise aversion (Dale et al., 2010; Blackwell et al., 2013; Tiira et al., 2013; Ballantyne, 2019; Gates et al., 2019; Gähwiler et al., 2020). This allowed us to pose questions to owners on specific behaviours such as panting and drooling as stress signs and fleeing and trembling as fear signs. After asking owners about these specific behaviours in their animals, we asked if their animal upon hearing firework exhibited stress and if their animal upon hearing firework exhibited fear. We added questions that we deemed relevant, but seemed previously unaddressed, such as the effect owners observed after (therapeutic) interventions directed at an animal's firework aversion. The questionnaire was pretested with two owners of cats and three of dogs to test for understanding and omissions. Respondents were asked to fill out the questionnaire with one particular cat or dog in mind. Quantitative questions were typically answered on a five-point Likert scale. An example is an animal's tendency to display fear or stress signs not at all (0), very mildly (1), mildly (2), strongly (3) to very strongly (4). Finally, we added questions to indicate applicability of possible negative quality of life effects and these were scored on a scale from 0 to 100.

### 2.3 Data preparation and statistical analysis

Data from Qualtrics® was gathered in Microsoft® Excel. Statistical analysis of the data was performed with SPSS® for Windows®, version 29 (SPSS Inc, Chicago, USA). Records that missed values on the question items regarding firework-related behaviour(al consequences, *N* = 482) were deleted from the dataset as these would prevent us from running our primary analyses, on firework aversion. Descriptive statistics were analysed for the variables and presented as percentages (counts). Next to presenting data over all categories, for further analysis we grouped answers, such as for an animal displaying behavioural signs as ‘yes’ by grouping mildly (2), strongly (3) and very strongly (4) and as ‘no’ by grouping not at all (0) and very mildly (1). We ran Chi-square tests to indicate differences in counts, regarding as significantly different P-values <0.05 and residuals |>2|. Next to Chi-square tests, we used Spearman's rank correlation test, regarding P-values <0.001 and r_s_>|0.2] as statistically significant.

## 3 Results

### 3.1 Animals, animal living environment and early life experiences

Our study sample of 3631 respondents consisted mainly of females (88.9 %, *N* = 3228 of 3631; see Appendix B.1 for all details on gender, ages and living situations). Most respondents filled out the questionnaire for their dog 82.9 % (*N* = 3009; 17.1 % for their cat, *N* = 622; see Appendix B.2 for details). Most of the animals were *not* known to have a hearing impairment (98.3 % of the cats, *N* = 610; 97.2 % of the dogs, *N* = 2924). Owners mostly indicated that their animal had no medical issue (58.3 %, *N* = 1753 of the dogs; 60.8 %, *N* = 378 of the cats). Of the cats 13.8 % (*N* = 86) and of the dogs 9.3 % (*N* = 280) were reported to live solely with their owner. Most respondents lived in a house with garden (84.5 %; *N* = 3070), with only 16.8 % (*N* = 611) indicating to live more rurally (see Appendix B.1 for details). Of the cats, none were kept exclusively outdoors, but most had some access to the outdoors (see Appendix B.1 for details). Owners indicated that 61.5 % (*N* = 2234) of the animals did *not* have a pedigree. Separately from pedigree status, we asked about the acquisition source of the animal and most were acquired from a breeder that kept the litter inside the home, as seen in Table 1.

**Table 1:** Acquisition source of the cat or dog. Acquisition source of the cats (*N* = 622) and dogs (*N* = 3009) as indicated by their owners (*N* = 3631 responses total).

	**% (N of total 622) for cats**	**% (N of total 3009) for dogs**	**% (N of total 3631) for both species**
Born with owner	3.2 % (20)	2.5 % (74)	2.6 % (94)
Breeder - litter kept in home environment	33.0 % (205)	45.7 % (1374)	43.5 % (1579)
Breeder - litter *not* kept in home environment	9.3 % (58)	16.1 % (484)	14.9 % (542)
Delivered at home	2.1 % (13)	0.3 % (10)	0.6 % (23)
Rehomed via organisation placing animals from *outside* the country	4.5 % (28)	21.6 % (649)	18.6 % (677)
Rehomed via organisation placing animals from *within* the country	30.2 % (188)	4.3 % (129)	8.7 % (317)
Rehomed via relative	10.5 % (65)	6.4 % (193)	7.1 % (258)
Don't know or other	7.2 % (45)	3.2 % (96)	3.9 % (141)

Of the early life factors, the factor of noise habituation by the breeder, was less common for cats (4.3 %; *N* = 27 of 622) than dogs (19.7 %; *N* = 593 of 3009 dogs). The highest percentage for habituation and socialisation aspects was for seeing more than two adults as a kitten or puppy: 63.5 % (*N* = 2307 animals), with a lower reporting for cats (50.8 %, *N* = 316) than dogs (66.2 %, *N* = 1991). Appendix B.3 lists the reported habituation and socialisation opportunities for cats and dogs. Generally cats seemed to receive such opportunities at lesser percentages than dogs.

### 3.2 Firework exposure

**Fig. 1:**
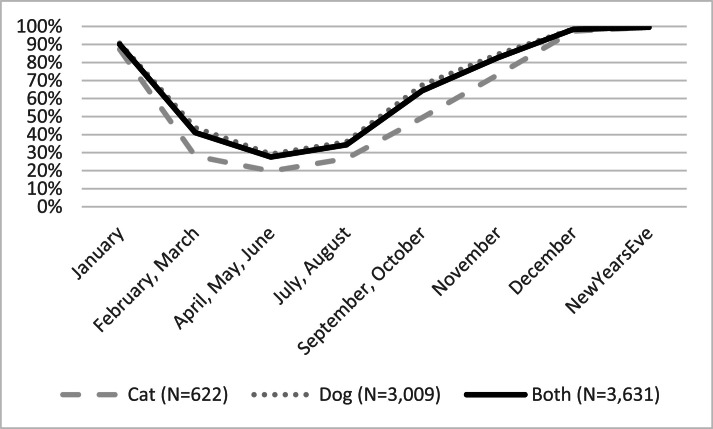
Percentage of respondents hearing firework noise in their environment during the months of the year and on New Year's Eve.

Firework noise was reportedly heard in the respondents’ living environments from September onwards by 64.3 % of the respondents (*N* = 2335) and by 82.6 % (*N* = 2998) in November, 98.4 % (*N* = 3572) in December, with higher percentages for dog owners, than cat owners, as shown in Fig. 1 (and see Appendix C.1 for all details).

In periods of much firework noise, such as December, firework is heard more than three times daily by 78.4 % of the respondents (*N* = 2848; see Appendix C.2 for all details). Firework noise is logically described more loudly outdoors than inside the home environment (Appendix C.3). Yet, even indoors the noise may be heard loudly, for example ‘as loud as a thunder bolt within fifty meters’, by 20.1 % (*N* = 729) of the respondents. Firework noise was reported to have increased in the living environment of 59.8 % of the respondents in the past three years (*N* = 2170; no change: 33.8 %, *N* = 1229; decrease: 6.1 %, *N* = 220; unknown: 0.3 %, *N* = 12).

Of the dog owning respondents, 60.5 % could walk their dog in a firework noise free area at maximum an hour distance from their home (*N* = 1814; at longer than an hour distance: 39.5 %, *N* = 1187; *N* = 8 missing values).

### 3.3 Firework reactions

During and/ or directly after firework most cats show the behaviours (mildly, strongly or very strongly; versus not showing these behaviours or showing these behaviours very mildly) of hiding (75.9 %, *N* = 472), fleeing (64.6 %, *N* = 402), freezing (42.8 %, *N* = 266) and seeking owner eye contact (55.6 %, *N* = 346) and/ or nearing the owner (43.4 %, *N* = 270) and most dogs of seeking owner eye contact (73.7 %, *N* = 2217), nearing the owner (68.9 %, *N* = 2073) and body contact with the owner (63.4 %, *N* = 1909), next to behaviours of trembling (63.5 %, *N* = 1912), and panting (60.2 %, *N* = 1810), (See Appendix D.1 for all details).

During a firework *period* (such as the week of New Years Eve) most cats show the behaviours (mildly, strongly or very strongly) of jumpiness (67.7 %, *N* = 421), hiding (66.7 %, *N* = 415), fleeing (63.7 %, *N* = 396), increased noise sensitivity (for other noise than firework; 52.6 %, *N* = 327), and moving their head skittishly (52.4 %, *N* = 326). For dogs also, the highest percentage was for the behaviour of jumpiness (69.0 %, *N* = 2076). Other behaviours during a firework period for dogs, were increased noise sensitivity (for other noise than firework; 59.1 %, *N* = 1777) and fleeing (57.6 %, *N* = 1734). Relatively higher percentages for dogs than cats were reported for owner attention/ nearness seeking (62.1 %, *N* = 1870) and separation related behaviour (57.4 %, *N* = 1728), with all details presented in Appendix D.2.

The often mentioned behaviours during a firework period can be seen as indicative of an animal feeling unsafe in its environment and it is therefore unsurprising that impaired welfare (mildly, strongly or very strongly) was reported for 69.5 % of the animals (*N* = 2525, cats: 63.5 %, *N* = 395; dogs: 70.8 %, *N* = 2130). ‘Unhappiness’ was indicated by 59.8 % of the owners (*N* = 2173, cats: 50.0 %, *N* = 311; dogs: 61.9 %, *N* = 1862).

In our study sample, 79.1 % (*N* = 2873) of the animals were indicated to experience stress because of firework and 77.3 % (*N* = 2807) fear. Owners indicated lesser frequencies of longer experienced stress durations for cats than dogs and dogs seemed resistant to going outdoors more than cats, as shown in Table 2.

**Table 2:** Owners indicating a cat or dog to experience stress and/ or fear (mildly, strongly, very strongly) as a consequence of the animal hearing firework. Owners indicating that their cat (*N* = 622) or dog (*N* = 3009) experiences one or more forms of firework aversion (mildly, strongly or very strongly; *N* = 3631 responses total).

	**% (N of total 622) for cats**	**% (N of total 3009) for dogs**	**% (N of total 3631) for both species**
Stress experience	78.1 % (486)	79.3 % (2387)	79.1 % (2873)
Fear experience during firework noise	76.8 % (478)	77.4 % (2329)	77.3 % (2807)
Fear for up to half an hour after firework noise	53.1 % (330)	59.8 % (1799)	58.6 % (2129)
Resistance to going outdoors after firework noise	33.8 % (210)	62.6 % (1885)	57.7 % (2095)
Resistance to going outdoors regardless of hearing firework noise, during a firework period	27.3 % (170)	55.5 % (1671)	50.7 % (1841)
Fear for longer than half an hour after hearing firework noise	34.2 % (213)	42.9 % (1291)	41.4 % (1504)
Phobic fear after hearing firework noise	14.1 % (88)	33.9 % (1019)	30.5 % (1107)

As an ultimate consequence of firework, 5.9 % (*N* = 213) of the respondents indicated that their animal was ever lost after hearing firework, such as through fleeing. Cats were reportedly lost more so than dogs (cats: 8.4 %, *N* = 52; dogs: 5.4 %, *N* = 161).

A *negative* influence on quality of life of the animal's firework aversion (at the moment of filling out the questionnaire), was scored on a scale from 0 to 100, with 0 meaning no negative influence and 100 meaning the maximum negative influence on quality of life. The mean (±SD) was 62.7 ± 33.6 (range 0 to 100) for quality of life for the animals, 52.3 ± 32.5 (range 0 to 100) for quality of life of the owners and 49.4 ± 32.7 (range 0 to 100) for quality of life of others in their environment. The percentages were higher for dogs (69.7 ± 31.4, 56.9 ± 31.8, 51.1 ± 32.5; all ranges 0–100) than cats (58.6 ± 31.1, 50.4 ± 31.1, 45.9 ± 31.8; all ranges 0–100).

### 3.4 Firework aversion and associations with early life of the animal

Early life of the animal was studied for several early life factors for both cats and dogs. (Appendix E presents details.) To test our hypothesis that suboptimal early life factors come with higher prevalence of firework aversion, we compared the early life factors of acquisition source and noise habituation for the counts for three levels of intensity of firework fear (absent or very mild, versus mild, versus strong or very strong fear), with Chi square tests.

With regard to the acquisition channel, for the cats this was not seen to associate with firework fear (χ^2^=23.3, *P* = 0.06, df=14, *N* = 622). However, for the dogs, such associations were found. Chi square tests revealed lower counts of no or mild firework fear and higher counts for (very) strong fear for rehomed dogs (acquired via an organisation outside of or within the country, such as a shelter, χ2=176.4, *P* < 0.001, df=14, *N* = 3009). A reverse pattern was for the dogs acquired from a breeder keeping the litter in a home environment and for the dogs born with the current owner (χ2=176.4, *P* < 0.001, df=14, *N* = 3009).

With regard to being noise habituated, for the cats this was not seen to associate with firework fear (*P* = 0.2). Relatively few cat owners reported to know that a kitten was noise habituated (4.3 %, *N* = 27 of 622), as previously mentioned. For the dogs, relatively more owners reported to know that a puppy was noise habituated (19.7 %, *N* = 593 of 3009) and such habituation related to later in life higher counts for no or mild firework fear (χ^2^=122.4, *P* < 0.001, df=2, *N* = 3009).

### 3.5 Firework aversion and associations with owner opinions on guiding and training an animal

We asked owners about their (dis)agreement on a scale from 1 (completely disagree) to 5 (completely agree) with statements on (reacting to) a general animal's firework aversion, so not specifically for the own animal. Cat and dog owners were on average in agreement with statements that an animal when reacting to firework, can be offered verbal support (mean±SD, range: 4.3 ± 1.0, 1–5) and support through touch and/ or stroking (4.2 ± 1.1, 1–5). Table 3 indicates how differences for cat and dogs were minimal. An average neutral opinion was for an owner's reaction determining an animal's future firework reactions (3.3 ± 1.3, 1–5). An average disagreement was measured on the statement that parental animals need to be opted for which are not noise sensitive (2.0 ± 1.0, 1–5) and that a puppy or kitten needs to be opted for that was noise habituated in its early life (2.6 ± 1.2, 1–5), to be able to prevent later in life firework reactions. (Appendix F lists all statements presented to the respondents.)

**Table 3:** Owners’ opinions on statements about animal guidance and acquisition choices, in relation to firework aversion. Statements were presented to 3631 respondents for them to rate from completely disagree (1) to completely agree (5) and we present mean±SD (range) for cat owning respondents (622), dog owning respondents (3009) and for owners of both species together.

	**Cats (*N*=622)**	**Dogs (*N*=3009)**	**Both species (*N*=3631)**
	**Mean±SD (range)**	**Mean±SD (range)**	**Mean±SD (range)**
People should not give an animal attention when it reacts to firework with ***a startle***	2.4 ± 1.3 (1–5)	2.1 ± 1.3 (1–5)	2.2 ± 1.3 (1–5)
People should not give an animal attention when it reacts to firework with ***fear***	2.0 ± 1.2 (1–5)	1.7 ± 1.1 (1–5)	1.8 ± 1.1 (1–5)
You may offer verbal support to an animal when it reacts to firework	4.3 ± 0.9 (1–5)	4.3 ± 1.0 (1–5)	4.3 ± 1.0 (1–5)
You may verbally correct an animal when it reacts to firework	1.2 ± 0.6 (1–5)	1.2 ± 0.7 (1–5)	1.2 ± 0.6 (1–5)
You may offer support to an animal through touch and/ or stroking it when it reacts to firework	4.1 ± 1.0 (1–5)	4.2 ± 1.1 (1–5)	4.2 ± 1.1 (1–5)
An animal may seek physical contact with you when it reacts to firework with ***a startle***	4.7 ± 0.7 (1–5)	4.7 ± 0.7 (1–5)	4.7 ± 0.7 (1–5)
An animal may seek physical contact with you when it reacts to firework with ***fear***	4.8 ± 0.5 (1–5)	4.7 ± 0.7 (1–5)	4.8 ± 0.6 (1–5)
An animal may be comforted when it reacts to firework	4.2 ± 1.0 (1–5)	4.0 ± 1.2 (1–5)	4.0 ± 1.2 (1–5)
An animal may be punished for unwanted behaviour in reaction to firework	1.2 ± 0.6 (1–5)	1.2 ± 0.7 (1–5)	1.2 ± 0.7 (1–5)
The owner's reaction will determine how an animal will react to firework in the future	3.5 ± 1.1 (1–5)	3.3 ± 1.3 (1–5)	3.3 ± 1.3 (1–5)
If adopting or buying a puppy or kitten in The Netherlands, you have to opt for parental animals which are not noise sensitive, otherwise you will not be able to prevent firework reactions at a later age.	1.8 ± 0.9 (1–5)	2.0 ± 1.0 (1–5)	2.0 ± 1.0 (1–5)
If adopting or buying a puppy or kitten in The Netherlands, you have to opt for an animal that was noise habituated in its early life, otherwise you will not be able to prevent firework reactions at a later age.	2.4 ± 1.2 (1–5)	2.6 ± 1.3 (1–5)	2.6 ± 1.2 (1–5)

When testing with Spearman rank correlation tests, we found an inverse relationship between an animal's firework aversion scores and agreement with the statement ‘The owner's reaction will determine how an animal will react to firework in future’. Higher agreement with the statement related significantly to cats’ reported stress (r_s_=−0.23, *P* < 0.001) and fear (r_s_=−0.23, *P* < 0.001), explaining 5.3 % of variation in answers. For dogs up to 7.8 % of variation in answers was explained (stress: r_s_=−0.28, *P* < 0.001; fear: r_s_=−0.27, *P* < 0.001).

We then tested associations between the statements on attention, comfort and support-giving (via touching/stroking) and the behaviours owners reported their animals to show during firework. Interestingly, animals’ owner nearness-behaviours related differently for cats and dogs, as shown in Table 4. For cat owners, the statements on *attention-giving and comfort-giving* related to the animals’ owner nearness-behaviours. For dog owners, the statements on *support-giving* related to the animals’ owner nearness-behaviours (all r_s_>|0.2|, *P* < 0.001).

**Table 4:** Spearman rank correlations between owners’ opinions on animal guidance statements and the animal's owner-directed nearness behaviours. Statements were presented to 3631 respondents for them to rate from completely disagree (1) to completely agree (5) and animal behaviours were reported on by the same owners and we present Spearman rank correlations for r_s_>|0.2|, *P* < 0.001 for associations between statement agreement and animal behaviours’ reporting for cat owning respondents (*N* = 622) and dog owning respondents (*N* = 3009).

	**Being near the owner during firework noise**	**Bodily contact with owner during firework noise**	**Looking at the owner during firework noise**
People should not give an animal attention when it reacts to firework with ***a startle***	Cats: r_s_=−0.20, *P* < 0.001	Cats: r_s_=−0.24, *P* < 0.001	Cats: r_s_=−0.21, *P* < 0.001
People should not give an animal attention when it reacts to firework with ***fear***		Cats: r_s_=−0.23, *P* < 0.001	
An animal may be comforted when it reacts to firework		Cats: r_s_=0.20, *P* < 0.001	
You may offer support to an animal through touch and/ or stroking it when it reacts to firework	Dogs: r_s_=0.21, *P* < 0.001	Dogs: r_s_=0.22, *P* < 0.001	Dogs: r_s_=0.21, *P* < 0.001

For cat owners the cat's looking at the owner during firework (r_s_=0.21, *P* < 0.001) and being near the owner during firework (r_s_=0.20, *P* < 0.001) also related to an owner's higher agreement to the statement ‘My animal sees me as a source of support’, explaining up to 4.4 % of variation.

### 3.6 Owner advice seeking and interventions upon their animal's firework reactions

Dog owners reported to seek advice for their animal's firework reaction more (53.6 %, *N* = 1613) than cat owners (23.2 %, *N* = 144). Veterinarian professionals were most often mentioned amongst the sources of advice, for both species (26.1 %, 868 of *N* = 3331 times that advice was sought for animals, with multiple sources reported on for each animal). A seemingly large role was also for internet and social media (16.5 %, *N* = 548 of *N* = 3331 times advice sought for animals). Animal trainers, instructors, and coaches were consulted more for dogs (16.9 %, *N* = 525 of *N* = 3113 times advice sought for dogs) than cats (2.3 %, *N* = 5 of *N* = 218 times advice sought for cats), as were animal behavioural therapists (15.4 %, *N* = 479 of *N* = 3113 times advice sought for dogs; cats: 6.0 %, *N* = 13 of *N* = 218 times advice sought for cats). For cats, after the veterinarian profession and internet and social media, relatives were an often-consulted source of advice (15.1 %, *N* = 33 of *N* = 218 times advice sought for cats). See Appendix G.1 for all details. Multiple sources of advice could be consulted, as seen in a mean (± SD, range) 1.9 ± 1.2 (1–7) number of sources of advice mentioned, if advice was sought for an animal with firework aversion. For cats (1.5 ± 0.8, 1–4) this mean was lower than for dogs (1.9 ± 1.2, 1–7).

Of the 33 questioned (therapeutic) intervention options (excluding use of different garment or leash to prevent escape and the ‘other’ option, which held answers like ‘bringing the animal to care facility away from firework’, ‘clicker training for self-confidence’, ‘giving an animal eggnog liquor’), over both species a mean (± SD, range) 8.6 ± 6.9 (0–27) number of interventions was reportedly made by respondents for their animal's firework aversion. For cats (5.9 ± 4.5, 0–22) this mean was lower than for dogs (9.2 ± 7.2, 0–27).

Most frequently reported interventions for animals with firework aversion, were providing it with support and/ or comfort (67.5 %, *N* = 2452 for support and 61.7 %, *N* = 2241 for comfort), with little difference between owners’ opinions on support and comfort giving and between the two species (for all details see Appendix G.2). An example of providing support is in allowing an animal to stay close to the owner and an example of providing comfort is in talking to an animal in a soft voice. Providing hiding opportunity was the third most frequent intervention reported for cats (56.3 %, *N* = 350). For dogs this was limiting (masking) the noise of firework (58.2 %, *N* = 1752).

Lasting mitigating effects of firework aversion (therapeutic) interventions were below 30 % for the most often applied interventions, when grouping the responses of both species and relative to the number of times an intervention was applied. The highest percentage of a lasting effect was for relocating to an area with less environmental noise, such as a holiday home (29.4 %, *N* = 281 of 956 reported interventions). For the most often applied interventions of providing support/ comfort, a lasting effect was reported of slightly above 20 %. For cats, pheromone-based products, amongst the more often applied interventions, were indicated to have a lasting effect for only 6.1 % of the cats (*N* = 9 of 147). For the effect during one month, owners indicated for both species together at the highest percentages firstly behavioural medicine like benzodiazepine or gabapentin (Neurontin): 35.1 % (*N* = 105 of 299), secondly: relocating to an area with less environmental noise: 34.0 % (*N* = 326 of 956) and thirdly: behavioural medicine like clomipramine or dexmedetomidine: 26.3 % (*N* = 111 of 422). Appendix G.3 presents data for all answer options, which were a lasting effect, an effect for longer than a month, for the first month or no positive effect.

## 4 Discussion

We demonstrate in our explorative study on firework noise, based on a convenience sample of 622 Dutch cat owners and 3009 Dutch dog owners that firework affects a near two-thirds of animals from September onwards. Our respondents most frequently indicate a cat's behaviour of hiding, fleeing and seeking owner eye contact upon hearing firework and a dog's seeking owner eye contact, nearing the owner and trembling. This aligns our findings partially to previous studies that reported for cats with firework fear as most frequent behaviours: hiding, escaping or running away, but also trembling and cowering (Dale et al., 2010) and for dogs in one study: trembling, but also hiding and cowering (Dale et al., 2010) and in another study on loud noise, so not specifically firework: seeking out people and trembling, but also hiding and escaping (Blackwell et al., 2013).

We found high frequencies for an animal's owner nearness-behaviours, such as through eye contact. This finding may be based on differences in methodology, as contrary to Dale et al. (2010) and Blackwell et al. (2013) our questionnaire surveyed animal nearness-behaviours as part of the animal's firework aversion related behaviours. An animal's owner-directed behaviours, including nearness-behaviours, are of interest as animal owners are an important part of the (social and non-social) environment in which an animal is confronted with firework. Indeed, owners providing an animal with owner support and/ or comfort was amongst the highest frequencies of (therapeutic) interventions for firework aversion reported by owners in our study. Owner support/ comfort-giving can be hypothesized to counter stress experiences in animals with firework aversion, following theories of social buffering (Cohen & Wills, 1985; Hennessey et al., 2009). Although we know of no studies on social buffering and firework aversion, cats were seen to spend more time near a cat-attentive than non-attentive human (Vitale & Udell, 2019) and used referential looking to some degree, when confronted with a potentially scary novel object (Merola et al., 2015). Dogs in turn, benefited from a safe haven effect provided by their owner, when confronted with a threatening stranger, as reflected in lower heart rate increase in the presence of their owner, than without (Gácsi et al., 2013). Based on such findings a role for owner support/ comfort-giving may be hypothesized, although our study indicates that by itself these actions may not lead to lasting effects when used as an intervention for an animal suffering from firework aversion. Our respondents reported lasting effects for their support/ comfort-giving on an animal's firework aversion of slightly above 20 %. This was in line with similar low reporting (<30 %) for the other surveyed (therapeutic) interventions. Previous studies also find varying effectiveness-reporting for interventions addressing cat or dog firework aversion, ranging from pharmacological interventions through administering for example specific antidepressants or anxiolytics to behavioural strategies of the owner, such as ignoring an animal, systematic desensitisation or counterconditioning (Crowell-Davis et al., 2003; Sheppard & Mills, 2003; Levine et al., 2007; Cracknell & Mills, 2008; Levine & Mills, 2008; Sherman & Mills, 2008; Dale et al., 2010; Frank et al., 2010; Ballantyne, 2019; Engel et al., 2019; Gates et al., 2019; Morris et al., 2020; Riemer, 2020; Bergh et al., 2021). The seemingly low success rate of interventions once firework aversion affects an animal, highlights the importance of animal resilience to the development of firework aversion. Onset of firework aversion may be at a young age, as seen in 45 % of dogs suffering from firework fear already before the age of one year old (Riemer, 2019), further highlighting the need for ensuring resilience in (young) animals.

Such resilience will likely come from a favourable hereditary background and from noise habituation during the socialisation phases of cats and dogs (Blackwell et al., 2013; McMillan, 2014; Sonntag & Overall, 2014; Handegård, 2023). In our study we find an indication of a puppy's noise habituation possibly protecting the dog against later in life firework aversion, but not for cats for which such kitten noise-habituation was reported on at low frequencies in our study.

An importance of hereditary and early life factors suggests that animal owners opting for a kitten or puppy should be sufficiently aware of how these factors may affect an animal's later in life firework reactions. This makes it worrying that our respondents on average were not in (high) agreement with statements on the need to opt for a kitten or puppy that is noise habituated and whose parental animals are *not* noise sensitive, as to prevent later in life firework reactions. If indeed animal owners are insufficiently aware of how hereditary and early life factors may contribute to firework aversion, they may unknowingly make choices for young animals that come with lesser resilience and life-long animal welfare issues. This provides argumentation to study how to design effective educational interventions for cat and dog owners on the life-long influence of hereditary and early life factors. Generally, educational interventions for cat and dog owners seem understudied at present, but the complexity thereof was indicated. Studies addressed how owner to animal relationship characteristics may affect an owner's interpretation of animal behavioural signs (Bouma et al., 2024), how variation in response efficacy was seen for educational content (McLeod et al., 2017) and how a general variation in openness to learning from educational interventions may be relevant (Philpotts et al., 2019; Van Herwijnen, 2021).

Animal owners do not only affect hereditary background and early life situations of animals through their choice for a particular kitten or puppy. As stated before, the animal owner is also an element in the (social) environment of animals exposed to firework. Hence, although owner support/ comfort-giving may not be enough as an intervention to provide a lasting effect on firework aversion, it may be of some importance to the animal, depending on for instance an animal's early life or coping strategies. Additionally, we point out that owner support/ comfort-giving may be relevant to the owner. Owners of animals with firework aversion may experience a loss of control and predictability, as seen in owners reporting quality of life effects for themselves as well as for their animals. Being able to offer an animal support/ comfort may provide owners with some (restored) sense of control when confronted with their animal's firework aversion. The extent to which an animal seeks out owner support/ comfort may relate to the owner's behaviours. Owner behaviours in turn, may relate to owner views on appropriate handling if an animal reacts to firework noise. Indeed, we found a direct relationship between owners agreeing that an animal can be comforted/ supported and their animal's owner-directed contact behaviours and an inverse relationship between owners agreeing with a statement that animals that react to firework should *not* be given attention, yet not for both species for all statements.

We were also interested to learn if owners may provide themselves a different causal role, depending on an animal's firework aversion. We hypothesized that owners that report less firework aversion for their animal, agree more to the statement that owner behaviour affects an animal's future reactions to firework, following cognitive dissonance theory. This theory regards the prevention of mental discomfort by selectively processing environmental information (Harmon-Jones et al., 2015). Possibly a role for these mental processes exists as we found an inverse relationship between an animal's firework aversion scores and the agreement with a statement on an owner's reaction determining an animal's future firework reactions.

If such processes affect owner-reporting on animal firework aversion, this highlights an even greater need for prospective data gathering. Our study was based on retrospective owner reporting. Consequently, bias such as of recall bias, can be expected between actual cat or dog behaviour and owner reporting thereof. We surveyed our respondents without offering instructions or training on (observing) animal behaviours and thus our study cannot be regarded as an accurate reflection of all firework aversion behaviours shown by cats and dogs. Our questionnaire was clearly addressing the topic of firework and this will have resulted in higher numbers of participants with an interest in the topic. Such interest may coincide with higher exposure to firework in the environment, possibly causing bias. Additionally, our study sample may consist of more highly engaged animal owners. We searched for demographics of the Dutch dog and cat owner population but were unable to find statistics that could be used for comparison with our study sample. We point out that our study sample is likely not representative for all Dutch dog and cat owners, as in our explorative study we worked with a lengthy questionnaire that is more likely filled out by more highly engaged companion animal owners willing to take the effort to partake in this type of research.

Our questionnaire responses were gathered in a time frame that participants reported an increased firework exposure, which may have affected responses. We also highlight the different participation rate for cats and dogs, which is lower for cat owners. We found early life factors to relate to firework fear later in life for the dogs, but not for the cats that were reported on. Although this may indicate a higher relevance of early life noise habituation to create firework noise resilient animals for dogs, alternative explanations are likely. For example, our lower number of people partaking for cats than dogs may have resulted in a lack of power to detect differences between early life factor exposure for the cats that were reported on. This as cats are known to become resilient to for instance veterinary visit stress or general handling based on early life factors (Karsh, 1983; McCune, 1995; Mariti et al., 2016). Finally, we point out that owners of cats may be identifying firework fear less in their animal than in the owners of dogs identify in their dogs. Such differences in identifying fear may be due to the behavioural signs shown by cats versus dogs. Also, a cat's behaviour may be less noticeable to an owner, and/ or less impactful on an owner's life. As an example, when Dale and colleagues (2010) asked owners to rank cats and dogs for firework fear severity on a Likert scale from minimum 1 to maximum 4, mean values differed significantly, although slightly (cats 2.6, dogs 2.9, *Z*=−5.6; *P* < 0.0001; Dale et al., 2010). In line with Dale and colleagues’ findings, our study indicates that studying owner perception of cat and dog behaviour may be relevant to learning how animal behaviour may or may not be perceived differently by companion animal owners, depending on the species showing the behaviour.

## 5 Conclusion

We indicate an importance of resilience in cats and dogs that will live in a noise rich environment, such as Dutch cities and towns, where loud noise can be heard daily and firework – although restricted – is heard often and close by their residences for multiple months a year, not just for the festive season. Such resilience may possibly come from choosing breeding pairs carefully as to prevent hereditary noise sensitivity and from ensuring adequate early life experiences. Our study highlights that presently cat and dog owners may not yet sufficiently recognise the importance of opting for resilient animals. This may come with animal welfare risks, as (therapeutic) interventions for firework aversion may not have lasting positive effects for animals and their owners. We indicate a need for prospective studies into the development of firework aversion in cats and dogs and effective prevention thereof. This need becomes overt by the duration that animals in The Netherlands suffer from firework aversion and the extent to which it affects animal welfare.

## CRediT authorship contribution statement

**Ineke R. van Herwijnen:** Conceptualization, Data curation, Formal analysis, Investigation, Methodology, Project administration, Validation, Visualization, Writing – original draft, Writing – review & editing. **Claudia M. Vinke:** Writing – review & editing, Methodology, Conceptualization. **Saskia S. Arndt:** Writing – review & editing. **Pascalle E.M. Roulaux:** Writing – review & editing, Writing – original draft, Visualization, Validation, Formal analysis, Data curation.

## Declaration of competing interest

The authors declare that they have no competing interests.

## Data Availability

The datasets used during the current study are available from the corresponding author on reasonable request.

## Appendix Supplementary materials

Image, application 1
