# Supplementary material for: Firework aversion in cats and dogs as reported by Dutch animal owners
Source: Vet Anim Sci. 2024 Oct 16;26:100402. doi: 10.1016/j.vas.2024.100402 (PMC11533647; doi:10.1016/j.vas.2024.100402)
Supplement: Supplementary file 1 [file mmc1.docx]

**Appendix A – Questionnaire sections and questions**

***Section 1 – Owner opinions on animal interactions***

How do you opinion on a scale from 1 (completely disagree) to 5 (completely agree) on the following statements regarding general animal reactions to firework situations?

*People should not give an animal attention when it reacts to firework with a startle*

*People should not give an animal attention when it reacts to firework with fear*

*You may offer verbal support to an animal when it reacts to firework*

*You may verbally correct an animal when it reacts to firework*

*You may offer support to an animal through touch and/ or stroking it when it reacts to firework*

*An animal may seek physical contact with you when it reacts to firework with a startle*

*An animal may seek physical contact with you when it reacts to firework with fear*

*An animal may be comforted when it reacts to firework*

*An animal may be punished for unwanted behaviour in reaction to firework*

*The owner’s reaction will determine how an animal will react to firework in future*

*If adopting or buying a puppy or kitten in The Netherlands, you have to opt for parental animals which are not noise sensitive, otherwise you will not be able to prevent firework reactions at a later age*

*If adopting or buying a puppy or kitten in The Netherlands, you have to opt for an animal that was noise habituated in its early life, otherwise you will not be able to prevent firework reactions at a later age*

How do you opinion on a scale from 1 (completely disagree) to 5 (completely agree) on the following statements regarding raising/ training your animal in general (outside of firework situations)?

*My animal is with me, because it is useful, such as by keeping mice away as a cat or by guarding as a dog*

*My animal must be able to show behaviour that is normal for its species*

*My animal must not behave undesirably towards people*

*My animal must not behave undesirably towards other animals*

*My animal must not behave undesirably towards me*

*My animal must listen to/ obey me*

*My animal must be able to fulfil its needs*

*My animal makes me proud*

*My animal provides me with company*

*My animal listens to/ obeys me*

*My animal leads a good life*

*My animal is happy*

*My animal can function without me*

*My animal sees me as a source of support*

*My animal is safe in its surroundings*

*My animal feels safe in its surroundings*

*My animal feels dependent upon me*

***Section 2 – The animal, its living environment, and its early life experiences***

Is your animal a dog or a cat?

What breed or type is your cat or dog?

What age is your cat or dog?

Since what age is your animal living with you?

Is your cat or dog female/ male/ neutered – chemically/ operatively?

Is your cat or dog (partially) deaf?

Is your cat or dog suffering from physical issues, if so, which?

Is your cat or dog living with other animals, human family members, if so, which?

What is your age and gender?

Is your cat or dog living with you indoors, outdoors, apartment/ home with/ without outdoor access?

For cat owners: does your cat have outdoor access, if so, which?

How would you describe your and your animal’s living environment?

How did you acquire your cat or dog?

Do you know if your cat or dog encountered the following before it was 16 weeks old: >2/ 5 different children, >2/ 5 different adults, travelling in a vehicle, being held >5 min daily, >15 min daily, >45 min daily, various play materials (toys, boxes, etc), various surfaces (for example tiled floor and garden tiles or concrete floor and grass), >2/ 5 different dogs, >1/ 3 different cats?

For dogs: did the breeder of your dog prepare your dog for hearing loud noises, such as to prevent gun shyness?

Do you know if you cat or dog in its first 7 weeks of life had a mother present in the same room with access to your animal?

Did you see the mother of your cat or dog? Did you see the mother of your cat or dog groom, lick, care for your animal when it was a kitten or puppy?

How would you characterize the mother of your cat or dog?

***Section 3 – Firework exposure***

How would you describe firework noise in your living environment – frequency, strength, change over time?

If firework is within audible distance of your home, how long does it continue for, on average?

For dog owners: Can you walk your dog in a firework noise free area within 30 min/ 60 min reach?

***Section 4 – Firework reactions***

To which degree does your animal (not) display directly during and/ or directly after firework the following behaviours?

Salivation, panting, licking a certain part of the body, trembling, freezing, hiding, fleeing, panicking, escape behaviours, growling (dog), hissing (cat), biting, scratching (cat), barking, meowing, urinating, defecating, seeking eye contact/ approach/ contact with you.

To which degree does the following (not) apply to your animal upon hearing firework? Experiencing stress, fear: during firework/ ≤30 minutes after/ >30 minutes after hearing firework. Experiencing phobic fear, resistance to go outdoors: after hearing firework/ during firework periods.

To which degree does a firework period (such as the week of New Years Eve) (not) result in following behaviours in your animal: urinating/ defecating in inappropriate places, eating/ drinking more/ less, more licking/ biting (>5 sec in one place), pacing indoors, destructive behaviours, vocalisation, lethargy, less play, less seeking you out or your attention, more seeking you out or your attention, separation related behaviours, aggression such as growling, snapping (dog) or scratching (cat), being jumpy/ skittish, cowering, fleeing, hiding, reacting to other noises than firework, vomiting, defecating often or differently (including diarrhoea).

To which degree does the following (not) apply for our animal during a firework period (such as the week of New Years Eve)? Welfare impairment, unhappiness.

Was your animal ever lost after hearing firework, such as through fleeing?

When did you first see a firework reaction in your animal that resembled fear?

If you saw a first fear reaction in your animal, how did this originate?

If your animal ever was confronted with a firework incident (experiencing firework close by and resulting in an aversive reaction afterward), did it result in fear for more than firework (later on)?

If your animal reacts to firework, did you consult someone, if so, whom?

If your animal reacts to firework, did you attempt any interventions, if so, which? Did it have an effect, if so, which? Were you satisfied with the intervention, if so, how satisfied?

Do you take any preventive measures to prevent firework aversion? If so, which?

At the moment of filling out this questionnaire, if you experience a negative influence of your animal’s firework aversion, how would you rate this on a scale from 0-100 for influence on quality of life for your animal, yourself and for others in your environment?

**Appendix B.1 – Human respondent data and living situations**

|  | **% (N of total 622) for cat owners** | **% (N of total 3,009) for dog owners** |
| --- | --- | --- |
| *Gender* |  |  |
| Other of prefer not to say | 2.2% (14) | 1.4% (41) |
| Female | 88.4% (550) | 89.0% (2,678) |
| Male | 9.3% (58) | 9.6% (290) |
| *Age* |  |  |
| 18-35 years | 26.7% (166) | 14.7% (443) |
| 35-50 years | 33.6% (209) | 26.2% (788) |
| 50-65 years | 32.6% (203) | 45.3% (1,362) |
| >65 years | 6.8% (42) | 13.8% (415) |
| Prefer not to say | 0.3% (2) | 0% (1) |
| *Housing type* |  |  |
| Apartment with balcony | 21.5% (134) | 11.0% (331) |
| Apartment without balcony | 2.6% (16) | 1.0% (30) |
| Home with garden | 73.3% (456) | 86.9% (2,614) |
| Home without garden | 2.1% (13) | 0.8% (24) |
| Studio (one room only) | 0.5% (3) | 0.3% (10) |
| *Housing surroundings* |  |  |
| Village or suburb type | 36.7% (228) | 42.1% (1,527) |
| Inner city type, busy | 13.3% (83) | 10.2% (370) |
| Inner city type, quiet | 39.5% (246) | 30.9% (1,123) |
| Rural type, busy | 5.0% (31) | 7.0% (256) |
| Rural type, quiet | 5.5% (34) | 9.8% (355) |
| *Cat’s outdoor access type* |  |  |
| Outdoors as pleases, e.g. cat flap | 30.1% (187) |  |
| Outdoors when owner decides, not on leash | 35.2% (219) |  |
| Outdoors, on leash | 8.7% (54) |  |
| Outdoors only | 0.0% (0) |  |
| Indoors only | 25.6% (159) |  |
| Missing value | 0.5% (3) |  |

**Appendix B.2 – Animal characteristics**

|  | **% (N of total N=622) for cats** | **% (N of total N=3,009) for dogs** |
| --- | --- | --- |
| *Pedigree or non-pedigree* |  |  |
| Pedigree | 15.9% (99) | 43.1% (1,296) |
| Non-pedigree | 84.1% (523) | 56.9% (1,711) |
| *Age at time of participation* |  |  |
| Age <0.5 years | 1.0% (6) | 0.6% (18) |
| Age 0.5-1.5 years | 4.3% (27) | 3.8% (115) |
| Age 1.5-3 years | 11.7% (73) | 17.1% (515) |
| Age 3-10 years | 46.6% (290) | 58.0% (1,745) |
| Age 10-15 years | 28.0% (174) | 19.7% (593) |
| Age >15 years | 8.4% (52) | 0.8% (23) |
| *Age at acquisition by owner* |  |  |
| Age <16 weeks | 46.8% (291) | 62.2% (1,872) |
| Age 16 weeks-0.5 years | 18.6% (116) | 10.8% (326) |
| Age 0.5-1.5 years | 11.7% (73) | 10.6% (319) |
| Age 1.5-3 years | 7.9% (49) | 7.8% (234) |
| Age 3-10 years | 11.6% (72) | 7.9% (238) |
| Age 10-15 years | 3.1% (19) | 0.7% (20) |
| Age >15 years | 0.3% (2) | 0.0% (0) |
| *Neutering status and sex* |  |  |
| Female intact | 1.6% (10) | 10.1% (303) |
| Female (chemically or operatively) neutered | 46.6% (290) | 42.3% (1,270) |
| Male intact | 1.1% (7) | 18.0% (529) |
| Male (chemically or operatively) neutered | 50.6% (315) | 29.7% (894) |
| *Indoor or outdoor keeping* |  |  |
| Indoor only | 42.0% (261) | 58.3% (1,754) |
| Outdoor only | 0.0% (0) | 0.3% (10) |
| Indoor and outdoor | 58.0% (361) | 41.3% (1,242) |
| *Hearing impairment* |  |  |
| No hearing impairment known | 98.3% (610) | 97.2% (2,924) |
| Partially deaf | 1.3% (8) | 2.7% (81) |
| Completely deaf | 0.6% (4) | 0.1% (4) |

**Appendix B.3 – Reporting on habituation and socialisation opportunities of cats and dogs as kittens and puppies**

|  | **% (N of total 622) for cats** | **% (N of total 3,009) for dogs** |
| --- | --- | --- |
| *More than 2 children* |  |  |
| Yes | 33.0% (205) | 53.5% (1,609) |
| No | 17.7% (110) | 8.2% (246) |
| Unknown | 49.4% (307) | 38.4% (1,154) |
| *More than 5 children* |  |  |
| Yes | 7.4% (46) | 26.7% (802) |
| No | 34.4% (214) | 21.9% (659) |
| Unknown | 58.2% (362) | 51.4% (1,548) |
| *More than 2 adults* |  |  |
| Yes | 50.8% (316) | 66.2% (1,991) |
| No | 6.4% (40) | 3.2% (97) |
| Unknown | 42.8% (266) | 30.6% (921) |
| *More than 5 adults* |  |  |
| Yes | 24.4% (152) | 49.4% (1,487) |
| No | 19.3% (120) | 9.3% (280) |
| Unknown | 56.3% (350) | 41.3% (1,242) |
| *Travel in a vehicle* |  |  |
| Yes | 33.8% (210) | 55.6% (1,672) |
| No | 17.2% (107) | 8.7% (261) |
| Unknown | 49.0% (305) | 35.8% (1,076) |
| *Held for at least 5 minutes per day* |  |  |
| Yes | 48.6% (302) | 60.5% (1,819) |
| No | 3.5% (22) | 3.0% (89) |
| Unknown | 47.9% (298) | 36.6% (1,101) |
| *Held for at least 15 minutes per day* |  |  |
| Yes | 36.2% (225) | 47.8% (1,437) |
| No | 6.8% (42) | 6.4% (193) |
| Unknown | 57.1% (355) | 45.8% (1,379) |
| *Held for at least 45 minutes per day* |  |  |
| Yes | 17.5% (109) | 23.3% (700) |
| No | 14.5% (90) | 15.7% (473) |
| Unknown | 68.0% (423) | 61.0% (1,836) |
| *Presence of play material* |  |  |
| Yes | 52.6% (327) | 63.0% (1,897) |
| No | 2.7% (17) | 3.2% (95) |
| Unknown | 44.7% (278) | 33.8% (1,017) |
| *Several surfaces* |  |  |
| Yes | 43.1% (268) | 65.7% (1,977) |
| No | 8.0% (50) | 2.6% (78) |
| Unknown | 48.9% (304) | 31.7% (954) |
| *More than 2 other dogs* |  |  |
| Yes | 7.9% (49) | 57.6% (1,734) |
| No | 41.3% (257) | 8.9% (267) |
| Unknown | 50.8% (316) | 33.5% (1,008) |
| *More than 5 other dogs* |  |  |
| Yes | 1.3% (8) | 32.0% (962) |
| No | 47.6% (296) | 25.3% (762) |
| Unknown | 51.1% (318) | 42.7% (1,285) |
| *More than 1 other cat* |  |  |
| Yes | 44.5% (277) | 24.8% (746) |
| No | 10.6% (66) | 28.6% (862) |
| Unknown | 44.9% (279) | 46.6% (1,401) |
| *More than 3 other cats* |  |  |
| Yes | 21.7% (135) | 6.5% (197) |
| No | 28.6% (178) | 42.1% (1,267) |
| Unknown | 49.7% (309) | 51.3% (1,545) |
| *Noise habituation* |  |  |
| Yes | 4.3% (27) | 19.7% (593) |
| No | 48.2% (300) | 32.3% (972) |
| Unknown | 45.3% (282) | 45.4% (1,367) |
| *Mother seen (to care)* |  |  |
| Seen and seen to care | 27.0% (168) | 43.9% (1,322) |
| Seen but not to care | 7.2% (45) | 11.6% (348) |
| Not seen | 34.6% (215) | 21.0% (632) |
| Unknown | 31.2% (194) | 23.5% (707) |
| *Mother close* |  |  |
| Close with access | 40.5% (252) | 56.7% (1,706) |
| Close without access (partially) | 1.1% (7) | 2.8% (85) |
| Not close | 5.9% (37) | 1.9% (57) |
| Unknown | 52.4% (326) | 38.6% (1,161) |

**Appendix C.1 - Time of year that firework noise is heard**

|  | **% (N of total 622) for cats** | **% (N of total 3,009) for dogs** | **% (N of total 3,631) for both species** |
| --- | --- | --- | --- |
| *New Year’s Eve* |  |  |  |
| No | 0.5% (3) | 0.5% (16) | 0.5% (19) |
| Yes | 99.5% (619) | 99.5% (2,993) | 99.5% (3,612) |
| *January* |  |  |  |
| No | 12.7% (79) | 9.3% (280) | 9.9% (359) |
| Yes | 87.3% (543) | 90.7% (2,729) | 90.1% (3,272) |
| *February, March* |  |  |  |
| No | 71.9% (447) | 56.2% (1,691) | 58.9% (2,138) |
| Yes | 28.1% (175) | 43.8% (1,318) | 41.1% (1,493) |
| *April, May, June* |  |  |  |
| No | 80.2% (499) | 70.8% (2,130) | 72.4% (2,629) |
| Yes | 19.8% (123) | 29.2% (879) | 27.6% (1,002) |
| *July, August* |  |  |  |
| No | 73.3% (456) | 64.0% (1,927) | 65.6% (2,383) |
| Yes | 26.7% (166) | 36.0% (1,082) | 34.4% (1,248) |
| *September, October* |  |  |  |
| No | 50.6% (315) | 32.6% (981) | 35.7% (1,296) |
| Yes | 49.4% (307) | 67.4% (2,028) | 64.3% (2,335) |
| *November* |  |  |  |
| No | 26.8% (167) | 15.5% (466) | 17.4% (633) |
| Yes | 73.2% (455) | 84.5% (2,543) | 82.6% (2,998) |
| *December* |  |  |  |
| No | 2.6% (16) | 1.4% (43) | 1.6% (59) |
| Yes | 97.4% (606) | 98.6% (2,966) | 98.4% (3,572) |

**Appendix C.2 – Frequency and duration of hearing firework**

|  | **% (of total 622) for cats** | **N for cats** | **% (of total 3,009) for dogs** | **N for dogs** | **% (of total 3,631) for both species** | **N for both species** |
| --- | --- | --- | --- | --- | --- | --- |
| ***How often during low firework period*** |  |  |  |  |  |  |
| 1-3 per month or less | 65.0% | 404 | 56.6% | 1,703 | 58.0% | 2,107 |
| ≥1 per week, not daily | 27.5% | 171 | 32.9% | 991 | 32.0% | 1,162 |
| Daily, 1-3 times | 4.7% | 29 | 6.5% | 197 | 6.2% | 226 |
| Daily, 3-5 times | 2.1% | 13 | 2.1% | 64 | 2.1% | 77 |
| Daily, 5-10 times | 0.6% | 4 | 1.2% | 36 | 1.1% | 40 |
| Daily, 10 times or more | 0.2% | 1 | 0.6% | 18 | 0.5% | 19 |
| ***How often during high firework period*** |  |  |  |  |  |  |
| 1-3 per month or less | 1.9% | 12 | 3.1% | 92 | 2.9% | 104 |
| ≥1 per week, not daily | 7.6% | 47 | 8.9% | 268 | 8.7% | 315 |
| Daily, 1-3 times | 9.6% | 60 | 10.1% | 304 | 10.0% | 364 |
| Daily, 3-5 times | 14.8% | 92 | 15.9% | 479 | 15.7% | 571 |
| Daily, 5-10 times | 23.3% | 145 | 19.5% | 588 | 20.2% | 733 |
| Daily, 10 times or more | 42.8% | 266 | 42.5% | 1,278 | 42.5% | 1,544 |
| ***Duration when firework is heard*** |  |  |  |  |  |  |
| ≤1 minute | 32.5% | 202 | 22.0% | 662 | 23.8% | 864 |
| 1-2 minutes | 27.2% | 169 | 26.5% | 797 | 26.6% | 966 |
| 2-5 minutes | 23.8% | 148 | 25.3% | 761 | 25.0% | 909 |
| 5-15 minutes | 9.2% | 57 | 14.7% | 443 | 13.8% | 500 |
| 15-30 minutes | 3.7% | 23 | 5.9% | 179 | 5.6% | 202 |
| 30-60 minutes | 1.6% | 10 | 2.1% | 64 | 2.0% | 74 |
| ≥1 hour | 2.1% | 13 | 3.4% | 103 | 3.2% | 116 |

**Appendix C.3 – Indoors and outdoors hearing of firework**

|  | **% (of total 622) for cats** | **N for cats** | **% (of total 3,009) for dogs** | **N for dogs** | **% (of total 3,631) for both species** | **N for both species** |
| --- | --- | --- | --- | --- | --- | --- |
| ***Indoors hearing of firework*** |  |  |  |  |  |  |
| Do not hear anything | 0.3% | 2 | 0.6% | 17 | 0.5% | 19 |
| Alike soft whisper/ refrigerator hum | 2.7% | 17 | 4.3% | 129 | 4.0% | 146 |
| Alike normal conversation volume | 18.8% | 117 | 21.2% | 639 | 20.8% | 756 |
| Alike vacuum cleaner within a meter | 17.4% | 108 | 15.0% | 452 | 15.4% | 560 |
| Alike a hand dryer or hair dryer within a meter | 13.2% | 82 | 11.4% | 343 | 11.7% | 425 |
| Alike a food blender/ pneumatic drill within a meter | 17.5% | 109 | 16.3% | 489 | 16.5% | 598 |
| Alike a train passing by a platform at full speed/ car horn at five meters | 6.9% | 43 | 6.0% | 180 | 6.1% | 223 |
| Alike a loud entertainment venue, such as nightclubs, bars and concerts/ ambulance siren | 5.9% | 37 | 3.9% | 116 | 4.2% | 153 |
| Alike thunder bolt within 50 meters | 17.0% | 106 | 20.7% | 623 | 20.1% | 729 |
| Alike a plane taking off within 30 meters | 0.2% | 1 | 0.7% | 21 | 0.6% | 22 |
| *Outdoors hearing of firework* | |  |  |  |  |  |
| Do not hear anything | 0.5% | 3 | 0.0% | 0 | 0.1% | 3 |
| Alike soft whisper/ refrigerator hum | 0.3% | 2 | 0.3% | 8 | 0.3% | 10 |
| Alike normal conversation volume | 2.6% | 16 | 3.9% | 116 | 3.6% | 132 |
| Alike vacuum cleaner within a meter | 8.7% | 54 | 7.6% | 229 | 7.8% | 283 |
| Alike a hand dryer or hair dryer within a meter | 8.7% | 54 | 8.8% | 264 | 8.8% | 318 |
| Alike a food blender/ pneumatic drill within a meter | 19.3% | 120 | 18.4% | 553 | 18.5% | 673 |
| Alike a train passing by a platform at full speed/ car horn at five meters | 14.0% | 87 | 12.1% | 364 | 12.4% | 451 |
| Alike a loud entertainment venue, such as nightclubs, bars and concerts/ ambulance siren | 12.2% | 76 | 8.0% | 242 | 8.8% | 318 |
| Alike thunder bolt within 50 meters | 29.3% | 182 | 35.0% | 1,054 | 34.0% | 1,236 |
| Alike a plane taking off within 30 meters | 4.5% | 28 | 5.9% | 179 | 5.7% | 207 |

**Appendix D.1 – Behaviours of cats and dogs during or directly after hearing firework noise**

|  | **% (of total 622) for cats** | **N for cats** | **% (of total 3,009) for dogs** | **N for dogs** | **% (of total 3,631) for both species** | **N for both species** |
| --- | --- | --- | --- | --- | --- | --- |
| Support_eye contact | 55.6% | 346 | 73.7% | 2,217 | 70.6% | 2,563 |
| Support_nearing owner | 43.4% | 270 | 68.9% | 2,073 | 64.5% | 2,343 |
| Hiding | 75.9% | 472 | 58.5% | 1,760 | 61.5% | 2,232 |
| Support_body contact | 37.3% | 232 | 63.4% | 1,909 | 59.0% | 2,141 |
| Trembling | 35.5% | 221 | 63.5% | 1,912 | 58.7% | 2,133 |
| Fleeing | 64.6% | 402 | 52.1% | 1,568 | 54.3% | 1,970 |
| Panting | 8.0% | 50 | 60.2% | 1,810 | 51.2% | 1,860 |
| Freezing | 42.8% | 266 | 38.7% | 1,165 | 39.4% | 1,431 |
| Vocalizing | 12.2% | 76 | 31.9% | 959 | 28.5% | 1,035 |
| Panicking | 20.1% | 125 | 29.0% | 874 | 27.5% | 999 |
| Licking | 24.1% | 150 | 19.8% | 596 | 20.5% | 746 |
| Drooling | 3.9% | 24 | 22.5% | 677 | 19.3% | 701 |
| Breaking out | 11.7% | 73 | 16.7% | 502 | 15.8% | 575 |
| Growling | 9.6% | 60 | 15.1% | 455 | 14.2% | 515 |
| Defecating | 3.2% | 20 | 7.0% | 210 | 6.3% | 230 |
| Biting | 1.0% | 6 | 0.8% | 23 | 0.8% | 29 |
| Hissing_Cat | 8.8% | 55 |  |  |  |  |
| Scratching_Cat | 3.9% | 24 |  |  |  |  |
|  |  |  |  |  |  |  |

**Appendix D.2 – Behaviours of cats and dogs during periods of hearing firework noise**

|  | **% (of total 622) for cats** | **N for cats** | **% (of total 3,009) for dogs** | **N for dogs** | **% (of total 3,631) for both species** | **N for both species** |
| --- | --- | --- | --- | --- | --- | --- |
| Jumpiness/ spooked | 67.7% | 421 | 69.0% | 2,076 | 68.8% | 2,497 |
| Fleeing | 63.7% | 396 | 57.6% | 1,734 | 58.7% | 2,130 |
| More attention/ nearness seeking | 37.8% | 235 | 62.1% | 1,870 | 58.0% | 2,105 |
| Showing increased noise reaction (other noises) | 52.6% | 327 | 59.1% | 1,777 | 57.9% | 2,104 |
| Making jerky head moves | 52.4% | 326 | 53.9% | 1,623 | 53.7% | 1,949 |
| Hiding | 66.7% | 415 | 50.5% | 1,521 | 53.3% | 1,936 |
| Separation related behaviour | 32.5% | 202 | 57.4% | 1,728 | 53.2% | 1,930 |
| Restless behaviour indoors | 42.1% | 262 | 53.9% | 1,622 | 51.9% | 1,884 |
| Less playing | 30.5% | 190 | 40.3% | 1,214 | 38.7% | 1,404 |
| Walking with lower body (belly close to ground) | 44.9% | 279 | 36.6% | 1,101 | 38.0% | 1,380 |
| Vocalizing | 14.6% | 91 | 32.1% | 965 | 29.1% | 1,056 |
| Less water/ food intake | 21.5% | 134 | 29.7% | 894 | 28.3% | 1,028 |
| Lethargic or apathic behaviour | 17.8% | 111 | 20.8% | 626 | 20.3% | 737 |
| Less attention/ nearness seeking | 26.0% | 162 | 17.2% | 517 | 18.7% | 679 |
| Overly licking/ biting oneself (> 5 sec) | 15.8% | 98 | 17.2% | 518 | 17.0% | 616 |
| Soft stools/ diarrhoea and/ or increased frequency | 4.7% | 29 | 10.8% | 325 | 9.7% | 354 |
| Defecating indoors (dog) or outside litterbox (cat) | 6.1% | 38 | 9.0% | 270 | 8.5% | 308 |
| More water/ food intake | 3.9% | 24 | 5.6% | 170 | 5.3% | 194 |
| Vomiting | 3.7% | 23 | 4.0% | 120 | 3.9% | 143 |
| Aggression | 3.2% | 20 | 3.8% | 114 | 3.7% | 134 |
| Destructive behaviour | 1.8% | 11 | 3.5% | 104 | 3.2% | 115 |

**Appendix E - Cat and dog early life factors and firework stress and/ or firework fear**

***Table 1 - Counts of dogs per reported acquisition channel for animals reportedly experiencing firework stress at various levels***

A dog’s reported experiencing of stress due to firework as absent or very mild (score 0 or 1), versus mild (score 2), versus strong or very strong (score 3 or 4) compared for its reported channels of acquisition. Chi-square tests for these frequencies were significant and we present counts (residuals), marking in bold the observed count that deviates (residual |≥2|) from expected counts (χ^2^=161.3, P<0.001, df=14, N=3,009).

|  | **Born at current owner** | **Rehomed - relative** | **Rehomed - organi-sation within country** | **Rehomed - organi-sation outside country** | **Breeder - litter not in home environ-ment** | **Breeder - litter in home environ-ment** | **Delivered** | **Unknown** |
| --- | --- | --- | --- | --- | --- | --- | --- | --- |
| **No or very mild firework stress** | **29 (3.5)** | 35 (-0.8) | 17 (-1.9) | **42 (-8.0)** | 109 (0.9) | **368 (5.0)** | 3 (0.6) | 19 (-0.2) |
| **Mild firework stress** | 8 (0.5) | 14 (-0.8) | 14 (0.7) | **41 (-2.3)** | 40 (-0.6) | **147 (2.0)** | 1 (0.1) | 7 (-0.6) |
| **Strong or very strong firework stress** | **37 (-2.1)** | 144 (0.7) | 98 (0.8) | **566 (5.1)** | 335 (-0.3) | **859 (-3.4)** | 6 (-0.4) | 70 (0.3) |

***Table 2 – Counts of dogs per reported acquisition channel for animals reportedly experiencing firework fear at various levels***

A dog’s reported experiencing of fear due to firework as absent or very mild (score 0 or 1), versus mild (score 2), versus strong or very strong (score 3 or 4) compared for its reported channels of acquisition. Chi-square tests for these frequencies were significant and we present counts (residuals), marking in bold the observed count that deviates (residual |≥2|) from expected counts (χ^2^=176.4, P<0.001, df=14, N=3,009).

|  | **Born at current owner** | **Rehomed - relative** | **Rehomed – organis-ation within country** | **Rehomed – organi-sation outside country** | **Breeder - litter not in home environ-ment** | **Breeder - litter in home environ-ment** | **Delivered** | **Unknown** |
| --- | --- | --- | --- | --- | --- | --- | --- | --- |
| **No or very mild fear** | **30 (3.2)** | 38 (-0.9) | **18 (-2.1)** | **45 (-8.4)** | 116 (0.6) | **410 (5.6)** | 3 (0.5) | 20 (-0.4) |
| **Mild fear** | 8 (0.8) | 14 (-0.5) | 16 (1.7) | 41 (-1.7) | 34 (-0.9) | 124 (1.1) | 2 (1.3) | 8 (0.0) |
| **Strong or very strong fear** | **36 (-2.1)** | 141 (0.6) | 95 (0.6) | **563 (5.4)** | 334 (0.0) | **840 (-3.6)** | 5 (-0.7) | 68 (0.2) |

***Table 3 – Counts of dogs for closeness of the mother animal at puppy age for animals reportedly experiencing firework stress at various levels***

A dog’s reported experiencing of stress due to firework as absent or very mild (score 0 or 1), versus mild (score 2), versus strong or very strong (score 3 or 4) compared for closeness of the mother animal at puppy age, when assessing the separate answers of a mother animal being close with access all day, being close without access all day, with the mother not close, with the mother possibly close (unsure) and unknown. Chi-square tests for these frequencies were significant and we present counts (residuals), marking in bold the observed count that deviates (residual |≥2|) from expected counts (χ^2^=128.7, P<0.001, df=8, N=3,009).

|  | **Close with access all day** | **Close without access all day** | **Not close** | **Unsure** | **Unknown** |
| --- | --- | --- | --- | --- | --- |
| **No or very mild stress** | **461 (5.8)** | 22 (1.1) | 8 (-1.1) | **22 (-3.3)** | **109 (-6.2)** |
| **Mild stress** | 175 (1.7) | 6 (-0.6) | 5 (-0.1) | 10 (-2.1) | 76 (-1.1) |
| **Strong or very strong stress** | **1,070 (-3.7)** | 57 (-0.4) | 44 (0.6) | **179 (2.5)** | **765 (3.8)** |

***Table 4 – Counts of dogs for closeness of the mother animal at puppy age for animals reportedly experiencing firework fear at various levels***

A dog’s reported experiencing of fear due to firework as absent or very mild (score 0 or 1), versus mild (score 2), versus strong or very strong (score 3 or 4) compared for closeness of the mother animal at puppy age, when assessing the separate answers of a mother animal being close with access all day, being close without access all day, with the mother not close, with the mother possibly close (unsure) and unknown. Chi-square tests for these frequencies were significant and we present counts (residuals), marking in bold the observed count that deviates (residual |≥2|) from expected counts (χ^2^=134.3, P<0.001, df=8, N=3,009).

|  | **Close with access all day** | **Close without access all day** | **Not close** | **Unsure** | **Unknown** |
| --- | --- | --- | --- | --- | --- |
| **No or very mild fear** | **510 (6.3)** | 19 (0.0) | 8 (-1.4) | **23 (-3.6)** | **120 (-6.5)** |
| **Mild fear** | 148 (0.7) | 7 (0.0) | 5 (0.1) | 13 (-1.0) | 74 (-0.5) |
| **Strong or very strong fear** | **1,048 (-3.9)** | 59 (0.0) | 44 (0.7) | **175 (2.4)** | **756 (3.8)** |

***Table 5 – Counts of dogs for care provision of the mother animal at puppy age for animals reportedly experiencing firework stress at various levels***

A dog’s reported experiencing of stress due to firework as absent or very mild (score 0 or 1), versus mild (score 2), versus strong or very strong (score 3 or 4) compared for care provision of the mother animal at puppy age, when assessing the separate answers of seeing a mother animal care for an infant animal, seeing the mother animal, but not seeing her care for an infant animal, not seeing the mother animal and unknown. Chi-square tests for these frequencies were significant and we present counts (residuals), marking in bold the observed count that deviates (residual |≥2|) from expected counts (χ^2^=143.9, P<0.001, df=6, N=3,009).

|  | **Seen to care** | **Seen, but not to care** | **Not seen** | **Unknown** |
| --- | --- | --- | --- | --- |
| **No or very mild stress** | **381 (6.5)** | 80 (1.0) | **76 (-4.8)** | **85 (-5.1)** |
| **Mild stress** | **145 (2.3)** | 30 (-0.3) | **39 (-2.4)** | 58 (-0.7) |
| **Strong or very strong stress** | **796 (-4.4)** | 238 (-0.4) | **517 (3.5)** | **564 (3.0)** |

***Table 6 – Counts of dogs for care provision of the mother animal at puppy age for animals reportedly experiencing firework fear at various levels***

A dog’s reported experiencing of fear due to firework as absent or very mild (score 0 or 1), versus mild (score 2), versus strong or very strong (score 3 or 4) compared for care provision of the mother animal at puppy age, when assessing the separate answers of seeing a mother animal care for an infant animal, seeing the mother animal, but not seeing her care for an infant animal, not seeing the mother animal and unknown. Chi-square tests for these frequencies were significant and we present counts (residuals), marking in bold the observed count that deviates (residual |≥2|) from expected counts (χ^2^=151.3, P<0.001, df=6, N=3,009).

|  | **Seen to care** | **Seen, but not to care** | **Not seen** | **Unknown** |
| --- | --- | --- | --- | --- |
| **No or very mild fear** | **422 (7.1)** | 87 (0.9) | **76 (-5.6)** | **95 (-5.1)** |
| **Mild fear** | 118 (0.9) | 27 (-0.3) | 49 (-0.4) | 53 (-0.7) |
| **Strong or very strong fear** | **782 (-4.4)** | 234 (-0.4) | **507 (3.3)** | **559 (3.2)** |

**Appendix F – Owners’ opinions on several aspects of their animal, its behaviour, training in general and on guiding and training an animal about firework aversion**

|  | **Cats (N=622)** | **Dogs (N=3,009)** | **Both species (N=3,631)** |
| --- | --- | --- | --- |
|  | **Mean±SD (range)** | **Mean±SD (range)** | **Mean±SD (range)** |
| People should not give an animal attention when it reacts to firework with a startle | 2.4±1.3 (1-5) | 2.1±1.3 (1-5) | 2.2±1.3 (1-5) |
| People should not give an animal attention when it reacts to firework with fear | 2.0±1.2 (1-5) | 1.7±1.1 (1-5) | 1.8±1.1 (1-5) |
| You may offer verbal support to an animal when it reacts to firework | 4.3±0.9 (1-5) | 4.3±1.0 (1-5) | 4.3±1.0 (1-5) |
| You may verbally correct an animal when it reacts to firework | 1.2±0.6 (1-5) | 1.2±0.7 (1-5) | 1.2±0.6 (1-5) |
| You may offer support to an animal through touch and/ or stroking it when it reacts to firework | 4.1±1.0 (1-5) | 4.2±1.1 (1-5) | 4.2±1.1 (1-5) |
| An animal may seek physical contact with you when it reacts to firework with a startle | 4.7±0.7 (1-5) | 4.7±0.7 (1-5) | 4.7±0.7 (1-5) |
| An animal may seek physical contact with you when it reacts to firework with fear | 4.8±0.5 (1-5) | 4.7±0.7 (1-5) | 4.8±0.6 (1-5) |
| An animal may be comforted when it reacts to firework | 4.2±1.0 (1-5) | 4.0±1.2 (1-5) | 4.0±1.2 (1-5) |
| An animal may be punished for unwanted behaviour in reaction to firework | 1.2±0.6 (1-5) | 1.2±0.7 (1-5) | 1.2±0.7 (1-5) |
| The owner’s reaction will determine how an animal will react to firework in future | 3.5±1.1 (1-5) | 3.3±1.3 (1-5) | 3.3±1.3 (1-5) |
| If adopting or buying a puppy or kitten in The Netherlands, you have to opt for parental animals which are not noise sensitive, otherwise you will not be able to prevent firework reactions at a later age. | 1.8±0.9 (1-5) | 2.0±1.0 (1-5) | 2.0±1.0 (1-5) |
| If adopting or buying a puppy or kitten in The Netherlands, you have to opt for an animal that was noise habituated in its early life, otherwise you will not be able to prevent firework reactions at a later age. | 2.4±1.2 (1-5) | 2.6±1.3 (1-5) | 2.6±1.2 (1-5) |
| My animal is with me, because it is useful, such as by keeping mice away as a cat or by guarding as a dog | 1.4±0.9 (1-5) | 1.7±1.1 (1-5) | 1.6±1.1 (1-5) |
| My animal must be able to show behaviour that is normal for its species | 4.3±1.1 (1-5) | 4.4±0.9 (1-5) | 4.4±1.0 (1-5) |
| My animal must not behave undesirably towards people | 3.6±1.2 (1-5) | 4.1±1.0 (1-5) | 4.0±1.1 (1-5) |
| My animal must not behave undesirably towards other animals | 3.4±1.1 (1-5) | 3.9±1.0 (1-5) | 3.8±1.0 (1-5) |
| My animal must not behave undesirably towards me | 3.7±1.1 (1-5) | 4.3±1.0 (1-5) | 4.2±1.0 (1-5) |
| My animal must listen to/ obey me | 2.9±1.1 (1-5) | 4.1±0.8 (1-5) | 3.9±1.0 (1-5) |
| My animal must be able to fulfil is needs | 4.4±1.1 (1-5) | 4.5±1.0 (1-5) | 4.4±1.1 (1-5) |
| My animal makes me proud | 4.4±0.9 (1-5) | 4.5±0.8 (1-5) | 4.5±0.8 (1-5) |
| My animal provides me with company | 4.9±0.4 (2-5) | 4.9±0.5 (1-5) | 4.9±0.4 (1-5) |
| My animal listens to/ obeys me | 3.5±1.1 (1-5) | 4.4±0.7 (1-5) | 4.2±0.9 (1-5) |
| My animal leads a good life | 4.7±0.5 (2-5) | 4.8±0.5 (1-5) | 4.8±0.5 (1-5) |
| My animal is happy | 4.6±0.6 (2-5) | 4.7±0.6 (1-5) | 4.7±0.6 (1-5) |
| My animal can function without me | 3.4±1.2 (1-5) | 3.3±1.2 (1-5) | 3.4±1.2 (1-5) |
| My animal sees me as a source of support | 4.4±0.7 (2-5) | 4.7±0.6 (1-5) | 4.6±0.6 (1-5) |
| My animal is safe in its surroundings | 4.8±0.4 (2-5) | 4.9±0.4 (1-5) | 4.8±0.4 (1-5) |
| My animal feels safe in its surroundings | 4.6±0.6 (1-5) | 4.6±0.7 (1-5) | 4.6±0.7 (1-5) |
| My animal feels dependent upon me | 3.4±1.0 (1-5) | 3.7±0.9 (1-5) | 3.7±0.9 (1-5) |

**Appendix G.1 – Owners’ advice seeking upon their animals’ firework reactions**

|  | **Cat % (of N=218, N times advice sought for cats)** | | **Dog % (of N=3,113, N times advice sought for dogs)** | | **Total % (of N=3,331, N times advice sought for animals)** | |
| --- | --- | --- | --- | --- | --- | --- |
| Internet, social media | 23.4% | 51 | 16.0% | 497 | 16.5% | 548 |
| Relatives | 15.1% | 33 | 10.3% | 321 | 10.6% | 354 |
| Animal store | 9.2% | 20 | 8.0% | 248 | 8.0% | 268 |
| Animal behavioural therapist | 6.0% | 13 | 15.4% | 479 | 14.8% | 492 |
| Animal trainer, instructor, coach | 2.3% | 5 | 16.9% | 525 | 15.9% | 530 |
| Veterinarian profession | 33.0% | 72 | 25.6% | 796 | 26.1% | 868 |
| Other | 11.0% | 24 | 7.9% | 247 | 8.1% | 271 |
|  |  | 218 |  | 3,113 |  | 3,331 |

**Appendix G.2 – Owners’ reporting of applying interventions for their animal’s firework reactions**

| **Intervention application** | **% for cats** | **N for cats** | **% for dogs** | **N for dogs** | **% for both species** | **N for both species** |
| --- | --- | --- | --- | --- | --- | --- |
| Acupuncture | 0.6% | 4 | 1.4% | 42 | 1.3% | 46 |
| Activity decrease | 4.2% | 26 | 11.3% | 340 | 10.1% | 366 |
| Activity increase | 29.4% | 183 | 40.7% | 1,226 | 38.8% | 1,409 |
| Behavioural medicine like benzodiazepine or gabapentin | 2.7% | 17 | 13.5% | 405 | 11.6% | 422 |
| Behavioural medicine like clomipramine or dexmedetomidine | 3.4% | 21 | 9.2% | 278 | 8.2% | 299 |
| Body wrap | 1.0% | 6 | 23.6% | 711 | 19.7% | 717 |
| Brain activity | 15.0% | 93 | 41.5% | 1,248 | 36.9% | 1,341 |
| Cannabis products | 1.3% | 8 | 9.4% | 282 | 8.0% | 290 |
| Correcting behaviour | 0.8% | 5 | 3.9% | 118 | 3.4% | 123 |
| Covering ears of animal | 0.8% | 5 | 7.1% | 214 | 6.0% | 219 |
| Distract with food | 32.5% | 202 | 43.1% | 1,296 | 41.3% | 1,498 |
| Distract with play | 37.0% | 230 | 48.0% | 1,444 | 46.1% | 1,674 |
| Flower blossom products | 8.5% | 53 | 24.9% | 749 | 22.1% | 802 |
| Food supplements | 8.0% | 50 | 18.2% | 547 | 16.4% | 597 |
| Food with calm claim | 3.2% | 20 | 2.4% | 71 | 2.5% | 91 |
| Going out only at specific times | 29.1% | 181 | 55.8% | 1,679 | 51.2% | 1,860 |
| Going out only in country side (dog only) |  |  | 39.6% | 1,193 |  |  |
| Going out only in garden | 13.8% | 86 | 29.0% | 874 | 26.4% | 960 |
| Homeopathic and/ or herbal products | 7.9% | 49 | 25.5% | 767 | 22.5% | 816 |
| Limit firework sight | 56.1% | 349 | 57.7% | 1,736 | 57.4% | 2,085 |
| Limit firework noise (masking) | 46.6% | 290 | 58.2% | 1,752 | 56.2% | 2,042 |
| Opportunity to hide | 56.3% | 350 | 46.4% | 1,397 | 48.1% | 1,747 |
| Opportunity to locate (access to room) | 52.4% | 326 | 50.4% | 1,516 | 50.7% | 1,842 |
| Pheromone based products | 23.6% | 147 | 18.7% | 563 | 19.6% | 710 |
| Practice with noise, not stepwise | 3.1% | 19 | 13.6% | 409 | 11.8% | 428 |
| Practice with noise, stepwise | 9.3% | 58 | 32.0% | 963 | 28.1% | 1,021 |
| Practicing relaxation | 6.3% | 39 | 26.1% | 784 | 22.7% | 823 |
| Providing comfort | 61.3% | 381 | 61.8% | 1,860 | 61.7% | 2,241 |
| Providing support | 66.1% | 411 | 67.8% | 2,041 | 67.5% | 2,452 |
| Relocating to area with less environmental noise | 3.1% | 19 | 31.1% | 937 | 26.3% | 956 |
| Touch technique | 1.4% | 9 | 7.6% | 229 | 6.6% | 238 |

**Appendix G.3 – Owners’ reporting on intervention effectiveness when applying interventions for their animal’s firework reactions**

| **Intervention effectiveness** | **% for cats** | **N for cats** | **% for dogs** | **N for dogs** | **% for both species** | **N for both species** | |
| --- | --- | --- | --- | --- | --- | --- | --- |
| *Homeopathic and/ or herbal products* |  |  |  |  |  |  |  |
| No positive effect | 59.2% | 29 | 65.1% | 499 | 64.7% | 528 |  |
| Positive effect first month | 22.4% | 11 | 18.3% | 140 | 18.5% | 151 |  |
| Positive effect longer than a month, not permanent | 8.2% | 4 | 11.9% | 91 | 11.6% | 95 |  |
| Lasting positive effect | 10.2% | 5 | 4.8% | 37 | 5.1% | 42 |  |
|  | 100.0% | 49 | 100.0% | 767 | 100.0% | 816 |  |
| *Flower blossom products* |  |  |  |  |  |  |  |
| No positive effect | 71.7% | 38 | 65.8% | 493 | 66.2% | 531 |  |
| Positive effect first month | 13.2% | 7 | 19.4% | 145 | 19.0% | 152 |  |
| Positive effect longer than a month, not permanent | 11.3% | 6 | 10.4% | 78 | 10.5% | 84 |  |
| Lasting positive effect | 3.8% | 2 | 4.4% | 33 | 4.4% | 35 |  |
|  | 100.0% | 53 | 100.0% | 749 | 100.0% | 802 |  |
| *Cannabis products* |  |  |  |  |  |  |  |
| No positive effect | 62.5% | 5 | 67.4% | 190 | 67.2% | 195 |  |
| Positive effect first month | 12.5% | 1 | 16.0% | 45 | 15.9% | 46 |  |
| Positive effect longer than a month, not permanent | 0.0% | 0 | 9.6% | 27 | 9.3% | 27 |  |
| Lasting positive effect | 25.0% | 2 | 7.1% | 20 | 7.6% | 22 |  |
|  | 100.0% | 8 | 100.0% | 282 | 100.0% | 290 |  |
| *Pheromone based products* |  |  |  |  |  |  |  |
| No positive effect | 74.1% | 109 | 71.9% | 405 | 72.4% | 514 |  |
| Positive effect first month | 13.6% | 20 | 17.4% | 98 | 16.6% | 118 |  |
| Positive effect longer than a month, not permanent | 6.1% | 9 | 8.5% | 48 | 8.0% | 57 |  |
| Lasting positive effect | 6.1% | 9 | 2.1% | 12 | 3.0% | 21 |  |
|  | 100.0% | 147 | 100.0% | 563 | 100.0% | 710 |  |
| *Food supplements* |  |  |  |  |  |  |  |
| No positive effect | 50.0% | 25 | 62.3% | 341 | 61.3% | 366 |  |
| Positive effect first month | 20.0% | 10 | 17.7% | 97 | 17.9% | 107 |  |
| Positive effect longer than a month, not permanent | 20.0% | 10 | 13.2% | 72 | 13.7% | 82 |  |
| Lasting positive effect | 10.0% | 5 | 6.8% | 37 | 7.0% | 42 |  |
|  | 100.0% | 50 | 100.0% | 547 | 100.0% | 597 |  |
| *Food with calm claim* |  |  |  |  |  |  |  |
| No positive effect | 60.0% | 12 | 77.5% | 55 | 73.6% | 67 |  |
| Positive effect first month | 15.0% | 3 | 12.7% | 9 | 13.2% | 12 |  |
| Positive effect longer than a month, not permanent | 5.0% | 1 | 7.0% | 5 | 6.6% | 6 |  |
| Lasting positive effect | 20.0% | 4 | 2.8% | 2 | 6.6% | 6 |  |
|  | 100.0% | 20 | 100.0% | 71 | 100.0% | 91 |  |
| *Behavioural medicine like clomipramine or dexmedetomidine* | |  |  |  |  |  |  |
| No positive effect | 58.8% | 10 | 49.9% | 202 | 50.2% | 212 |  |
| Positive effect first month | 35.3% | 6 | 25.9% | 105 | 26.3% | 111 |  |
| Positive effect longer than a month, not permanent | 0.0% | 0 | 15.6% | 63 | 14.9% | 63 |  |
| Lasting positive effect | 5.9% | 1 | 8.6% | 35 | 8.5% | 36 |  |
|  | 100.0% | 17 | 100.0% | 405 | 100.0% | 422 |  |
| *Behavioural medicine like benzodiazepine or gabapentin* | |  |  |  |  |  |  |
| No positive effect | 33.3% | 7 | 43.2% | 120 | 42.5% | 127 |  |
| Positive effect first month | 52.4% | 11 | 33.8% | 94 | 35.1% | 105 |  |
| Positive effect longer than a month, not permanent | 4.8% | 1 | 12.2% | 34 | 11.7% | 35 |  |
| Lasting positive effect | 9.5% | 2 | 10.8% | 30 | 10.7% | 32 |  |
|  | 100.0% | 21 | 100.0% | 278 | 100.0% | 299 |  |
| *Acupuncture* |  |  |  |  |  |  |  |
| No positive effect | 50.0% | 2 | 47.6% | 20 | 47.8% | 22 |  |
| Positive effect first month | 25.0% | 1 | 26.2% | 11 | 26.1% | 12 |  |
| Positive effect longer than a month, not permanent | 25.0% | 1 | 16.7% | 7 | 17.4% | 8 |  |
| Lasting positive effect | 0.0% | 0 | 9.5% | 4 | 8.7% | 4 |  |
|  | 100.0% | 4 | 100.0% | 42 | 100.0% | 46 |  |
| *Body wrap* |  |  |  |  |  |  |  |
| No positive effect | 16.7% | 1 | 62.7% | 446 | 62.3% | 447 |  |
| Positive effect first month | 50.0% | 3 | 24.8% | 176 | 25.0% | 179 |  |
| Positive effect longer than a month, not permanent | 16.7% | 1 | 6.5% | 46 | 6.6% | 47 |  |
| Lasting positive effect | 16.7% | 1 | 6.0% | 43 | 6.1% | 44 |  |
|  | 100.0% | 6 | 100.0% | 711 | 100.0% | 717 |  |
| *Touch technique* |  |  |  |  |  |  |  |
| No positive effect | 44.4% | 4 | 62.9% | 144 | 62.2% | 148 |  |
| Positive effect first month | 44.4% | 4 | 22.3% | 51 | 23.1% | 55 |  |
| Positive effect longer than a month, not permanent | 0.0% | 0 | 7.9% | 18 | 7.6% | 18 |  |
| Lasting positive effect | 11.1% | 1 | 7.0% | 16 | 7.1% | 17 |  |
|  | 100.0% | 9 | 100.0% | 229 | 100.0% | 238 |  |
| *Activity increase* |  |  |  |  |  |  |  |
| No positive effect | 57.9% | 106 | 62.3% | 764 | 61.7% | 870 |  |
| Positive effect first month | 20.8% | 38 | 18.8% | 230 | 19.0% | 268 |  |
| Positive effect longer than a month, not permanent | 8.2% | 15 | 9.2% | 113 | 9.1% | 128 |  |
| Lasting positive effect | 13.1% | 24 | 9.7% | 119 | 10.1% | 143 |  |
|  | 100.0% | 183 | 100.0% | 1,226 | 100.0% | 1,409 |  |
| *Activity decrease* |  |  |  |  |  |  |  |
| No positive effect | 84.6% | 22 | 77.9% | 265 | 78.4% | 287 |  |
| Positive effect first month | 11.5% | 3 | 10.9% | 37 | 10.9% | 40 |  |
| Positive effect longer than a month, not permanent | 0.0% | 0 | 6.2% | 21 | 5.7% | 21 |  |
| Lasting positive effect | 3.8% | 1 | 5.0% | 17 | 4.9% | 18 |  |
|  | 100.0% | 26 | 100.0% | 340 | 100.0% | 366 |  |
| *Brain activity* |  |  |  |  |  |  |  |
| No positive effect | 57.0% | 53 | 56.5% | 705 | 56.5% | 758 |  |
| Positive effect first month | 16.1% | 15 | 21.6% | 270 | 21.3% | 285 |  |
| Positive effect longer than a month, not permanent | 8.6% | 8 | 9.4% | 117 | 9.3% | 125 |  |
| Lasting positive effect | 18.3% | 17 | 12.5% | 156 | 12.9% | 173 |  |
|  | 100.0% | 93 | 100.0% | 1,248 | 100.0% | 1,341 |  |
| *Opportunity to hide* |  |  |  |  |  |  |  |
| No positive effect | 36.0% | 126 | 51.8% | 723 | 48.6% | 849 |  |
| Positive effect first month | 23.7% | 83 | 21.2% | 296 | 21.7% | 379 |  |
| Positive effect longer than a month, not permanent | 12.9% | 45 | 10.5% | 146 | 10.9% | 191 |  |
| Lasting positive effect | 27.4% | 96 | 16.6% | 232 | 18.8% | 328 |  |
|  | 100.0% | 350 | 100.0% | 1,397 | 100.0% | 1,747 |  |
| *Opportunity to locate (access to room)* |  |  |  |  |  |  |  |
| No positive effect | 35.9% | 117 | 52.4% | 794 | 49.5% | 911 |  |
| Positive effect first month | 22.4% | 73 | 19.2% | 291 | 19.8% | 364 |  |
| Positive effect longer than a month, not permanent | 11.3% | 37 | 10.5% | 159 | 10.6% | 196 |  |
| Lasting positive effect | 30.4% | 99 | 17.9% | 272 | 20.1% | 371 |  |
|  | 100.0% | 326 | 100.0% | 1,516 | 100.0% | 1,842 |  |
| *Providing support* |  |  |  |  |  |  |  |
| No positive effect | 38.7% | 159 | 42.0% | 858 | 41.5% | 1,017 |  |
| Positive effect first month | 21.9% | 90 | 20.7% | 423 | 20.9% | 513 |  |
| Positive effect longer than a month, not permanent | 11.7% | 48 | 13.9% | 283 | 13.5% | 331 |  |
| Lasting positive effect | 27.7% | 114 | 23.4% | 477 | 24.1% | 591 |  |
|  | 100.0% | 411 | 100.0% | 2,041 | 100.0% | 2,452 |  |
| *Providing comfort* |  |  |  |  |  |  |  |
| No positive effect | 40.2% | 153 | 46.0% | 855 | 45.0% | 1,008 |  |
| Positive effect first month | 23.1% | 88 | 20.3% | 377 | 20.7% | 465 |  |
| Positive effect longer than a month, not permanent | 12.6% | 48 | 13.0% | 241 | 12.9% | 289 |  |
| Lasting positive effect | 24.1% | 92 | 20.8% | 387 | 21.4% | 479 |  |
|  | 100.0% | 381 | 100.0% | 1,860 | 100.0% | 2,241 |  |
| *Correcting behaviour* |  |  |  |  |  |  |  |
| No positive effect | 60.0% | 3 | 71.2% | 84 | 70.7% | 87 |  |
| Positive effect first month | 0.0% | 0 | 13.6% | 16 | 13.0% | 16 |  |
| Positive effect longer than a month, not permanent | 0.0% | 0 | 7.6% | 9 | 7.3% | 9 |  |
| Lasting positive effect | 40.0% | 2 | 7.6% | 9 | 8.9% | 11 |  |
|  | 100.0% | 5 | 100.0% | 118 | 100.0% | 123 |  |
| *Distract with food* |  |  |  |  |  |  |  |
| No positive effect | 52.0% | 105 | 60.3% | 781 | 59.1% | 886 |  |
| Positive effect first month | 21.3% | 43 | 18.9% | 245 | 19.2% | 288 |  |
| Positive effect longer than a month, not permanent | 10.4% | 21 | 9.3% | 121 | 9.5% | 142 |  |
| Lasting positive effect | 16.3% | 33 | 11.5% | 149 | 12.1% | 182 |  |
|  | 100.0% | 202 | 100.0% | 1,296 | 100.0% | 1,498 |  |
| *Distract with play* |  |  |  |  |  |  |  |
| No positive effect | 57.8% | 133 | 60.8% | 878 | 60.4% | 1,011 |  |
| Positive effect first month | 17.4% | 40 | 18.8% | 271 | 18.6% | 311 |  |
| Positive effect longer than a month, not permanent | 10.0% | 23 | 8.4% | 122 | 8.7% | 145 |  |
| Lasting positive effect | 14.8% | 34 | 12.0% | 173 | 12.4% | 207 |  |
|  | 100.0% | 230 | 100.0% | 1,444 | 100.0% | 1,674 |  |
| *Limit firework sight* |  |  |  |  |  |  |  |
| No positive effect | 46.4% | 162 | 56.7% | 984 | 55.0% | 1,146 |  |
| Positive effect first month | 20.6% | 72 | 19.8% | 343 | 19.9% | 415 |  |
| Positive effect longer than a month, not permanent | 10.3% | 36 | 8.6% | 149 | 8.9% | 185 |  |
| Lasting positive effect | 22.6% | 79 | 15.0% | 260 | 16.3% | 339 |  |
|  | 100.0% | 349 | 100.0% | 1,736 | 100.0% | 2,085 |  |
| *Limit firework noise (masking)* |  |  |  |  |  |  |  |
| No positive effect | 45.2% | 131 | 52.5% | 919 | 51.4% | 1,050 |  |
| Positive effect first month | 23.8% | 69 | 22.0% | 386 | 22.3% | 455 |  |
| Positive effect longer than a month, not permanent | 10.0% | 29 | 10.2% | 178 | 10.1% | 207 |  |
| Lasting positive effect | 21.0% | 61 | 15.4% | 269 | 16.2% | 330 |  |
|  | 100.0% | 290 | 100.0% | 1,752 | 100.0% | 2,042 |  |
| *Covering ears of animal* |  |  |  |  |  |  |  |
| No positive effect | 80.0% | 4 | 67.3% | 144 | 67.6% | 148 |  |
| Positive effect first month | 0.0% | 0 | 15.0% | 32 | 14.6% | 32 |  |
| Positive effect longer than a month, not permanent | 0.0% | 0 | 8.9% | 19 | 8.7% | 19 |  |
| Lasting positive effect | 20.0% | 1 | 8.9% | 19 | 9.1% | 20 |  |
|  | 100.0% | 5 | 100.0% | 214 | 100.0% | 219 |  |
| *Relocating to area with less environmental noise* |  |  |  |  |  |  |  |
| No positive effect | 31.6% | 6 | 23.1% | 216 | 23.2% | 222 |  |
| Positive effect first month | 47.4% | 9 | 33.8% | 317 | 34.1% | 326 |  |
| Positive effect longer than a month, not permanent | 0.0% |  | 13.6% | 127 | 13.3% | 127 |  |
| Lasting positive effect | 21.1% | 4 | 29.6% | 277 | 29.4% | 281 |  |
|  | 100.0% | 19 | 100.0% | 937 | 100.0% | 956 |  |
| *Going out only in country side (dog only)* |  |  |  |  |  |  |  |
| No positive effect |  |  | 42.4% | 506 |  |  |  |
| Positive effect first month |  |  | 25.6% | 306 |  |  |  |
| Positive effect longer than a month, not permanent | |  | 12,2% | 146 |  |  |  |
| Lasting positive effect |  |  | 19.7% | 235 |  |  |  |
|  |  |  | 100.0% | 1,193 |  |  |  |
| *Going out only in garden* |  |  |  |  |  |  |  |
| No positive effect | 59.3% | 51 | 63.8% | 558 | 63.3% | 608 |  |
| Positive effect first month | 14.0% | 12 | 15.8% | 138 | 15.6% | 150 |  |
| Positive effect longer than a month, not permanent | 9.3% | 8 | 8.1% | 71 | 8.2% | 79 |  |
| Lasting positive effect | 17.4% | 15 | 12.2% | 107 | 12.7% | 122 |  |
|  | 100.0% | 86 | 100.0% | 874 | 100.0% | 960 |  |
| *Going out only at specific times* |  |  |  |  |  |  |  |
| No positive effect | 48.6% | 88 | 51.6% | 860 | 51.0% | 948 |  |
| Positive effect first month | 21.0% | 38 | 21.0% | 358 | 21.3% | 396 |  |
| Positive effect longer than a month, not permanent | 11.0% | 20 | 9.6% | 162 | 9.8% | 182 |  |
| Lasting positive effect | 19.3% | 35 | 17.8% | 299 | 18.0% | 334 |  |
|  | 100.0% | 181 | 100.0% | 1,679 | 100.0% | 1,860 |  |
| *Practice with noise, not stepwise* |  |  |  |  |  |  |  |
| No positive effect | 68.4% | 13 | 76.8% | 314 | 76.4% | 327 |  |
| Positive effect first month | 5.3% | 1 | 12.2% | 50 | 11.9% | 51 |  |
| Positive effect longer than a month, not permanent | 15.8% | 3 | 5.4% | 22 | 5.8% | 25 |  |
| Lasting positive effect | 10.5% | 2 | 5.6% | 23 | 5.8% | 25 |  |
|  | 100.0% | 19 | 100.0% | 409 | 100.0% | 428 |  |
| *Practice with noise, stepwise* |  |  |  |  |  |  |  |
| No positive effect | 50.0% | 29 | 65.4% | 630 | 64.5% | 659 |  |
| Positive effect first month | 17.2% | 10 | 14.1% | 136 | 14.3% | 146 |  |
| Positive effect longer than a month, not permanent | 12.1% | 7 | 8.1% | 78 | 8.3% | 85 |  |
| Lasting positive effect | 20.7% | 12 | 12.4% | 119 | 12.8% | 131 |  |
|  | 100.0% | 58 | 100.0% | 963 | 100.0% | 1,021 |  |
| *Practicing relaxation* |  |  |  |  |  |  |  |
| No positive effect | 43.6% | 17 | 57.8% | 453 | 57.1% | 470 |  |
| Positive effect first month | 23.1% | 9 | 18.4% | 144 | 18.6% | 153 |  |
| Positive effect longer than a month, not permanent | 15.4% | 6 | 9.6% | 75 | 9.8% | 81 |  |
| Lasting positive effect | 17.9% | 7 | 14.3% | 112 | 14.5% | 119 |  |
|  | 100.0% | 39 | 100.0% | 784 | 100.0% | 823 |  |
